# Synergistic interaction of Re complex and amine functionalized multiple ligands in metal-organic frameworks for conversion of carbon dioxide

**DOI:** 10.1038/s41598-017-00574-1

**Published:** 2017-04-04

**Authors:** Un Jin Ryu, Sang Jun Kim, Hyung-Kyu Lim, Hyungjun Kim, Kyung Min Choi, Jeung Ku Kang

**Affiliations:** 1 Graduate School of Energy, Environmental, Water and Sustainability (EEWS), Korea Advanced Institute of Science and Technology (KAIST), 291 Daehak-ro, Yuseong-gu, Daejeon 34141 Republic of Korea; 2 Department of Chemical and Biological Engineering, Sookmyung Women’s University, 100 Cheongpa-ro 47 gil, Yongsan-gu, Seoul 04310 Republic of Korea; 3 Department of Materials Science and Engineering, Korea Advanced Institute of Science and Technology (KAIST), 291 Daehak-ro, Yuseong-gu, Daejeon 34141 Republic of Korea

## Abstract

A metal-organic framework (MOF) is composed of secondary building units (SBUs) of metal ions and organic ligands to link each SBU. Moreover, the photosynthetic synthesis of a valuable CO chemical from carbon dioxide (CO_2_) represents an important class of appealing methods. Herein, we find that a molecular photocatalyst with high selectivity and activity can be designed via a fine balance in the proximity of Re complex (ReI(CO)_3_(BPYDC)(Cl), BPYDC = 2,2′-bipyridine-5,5′-dicarboxylate) and -NH_2_ functionalized multiple ligands composing a MOF photocatalyst, denoted as Re-MOF-NH_2_. These ligands in Re-MOF-NH_2_ has been confirmed by infrared, UV-visible, and ^1^H nuclear magnetic resonance spectra. Moreover, we show from extended X-ray absorption fine structure and *in*-*situ* infrared spectra that the bond corresponding to Re-CO upon introduction of -NH_2_ functional groups is divided into asymmetric bonds of 1.4 Å and 2.3 Å along with different CO_2_ vibrations, thus making the configuration of carbonyl groups in a Re metal complex become asymmetric in addition to aiding formation of CO_2_ intermediates within Re-MOF-NH_2_. Indeed, both of the uneven electron distribution in asymmetric carbonyl groups for Re-CO and the intermolecular stabilization of carbamate intermediates are proven to give the approximately 3-fold increase in photocatalytic activity for conversion of CO_2_ into CO.

## Introduction

Artificial photosynthesis using molecular catalysts^1–5^ is an attractive method for converting solar energy into value-added chemicals such as CO^6^. Hydrogen (H_2_) has a specific energy density much higher than those of conventional energy carriers while its nature in a gas state stable at ambient conditions, thus resulting in the storage issues in high pressures or extremely low temperatures. Meanwhile, a variety of liquefied fuels in principle, which are safe for transportation at ambient conditions, could be produced from CO on combination with gaseous H_2_ molecules^7^. For example, CO could be used to generate a liquefied fuel of methanol that has a high-energy density of 5,400 Wh per kilogram. Subsequently, methanol can be also used to produce other chemical products such as Olefin via “Methanol-to-Olefin (MTO)” process and Gasoline via “Methanol-to-Gasoline (MTG)” process^8^. Our recent work^9^ suggested a new strategy for tuning molecular photoactive ligand centres within the interior of a metal-organic framework (MOF) having embedded plasmon nanoparticles. Meanwhile, development of a molecular catalyst on combination of cooperative molecular ligands that enable the manipulation of the activity and selectivity for its photocatalytic conversion of CO_2_ into a valuable CO chemical with 100% selectivity would give a breakthrough solution to conversion of solar energy into useful hydrocarbon fuels, thus paving a new route for many promising applications. As of today, a single molecular catalyst with one kind of photoactive molecular ligand centres often does not have functional group enabling cooperation conversion of carbon dioxide into CO with high selectivity and activity. Additionally, absorption of visible light on the main solar energy spectrum is still limited for even molecular catalysts with multiple ligands. Therefore, it is advantageous for a molecular catalyst to have conjugated ligands enabling synergistic conversion of carbon dioxide upon visible light absorption.

**Figure 1:**
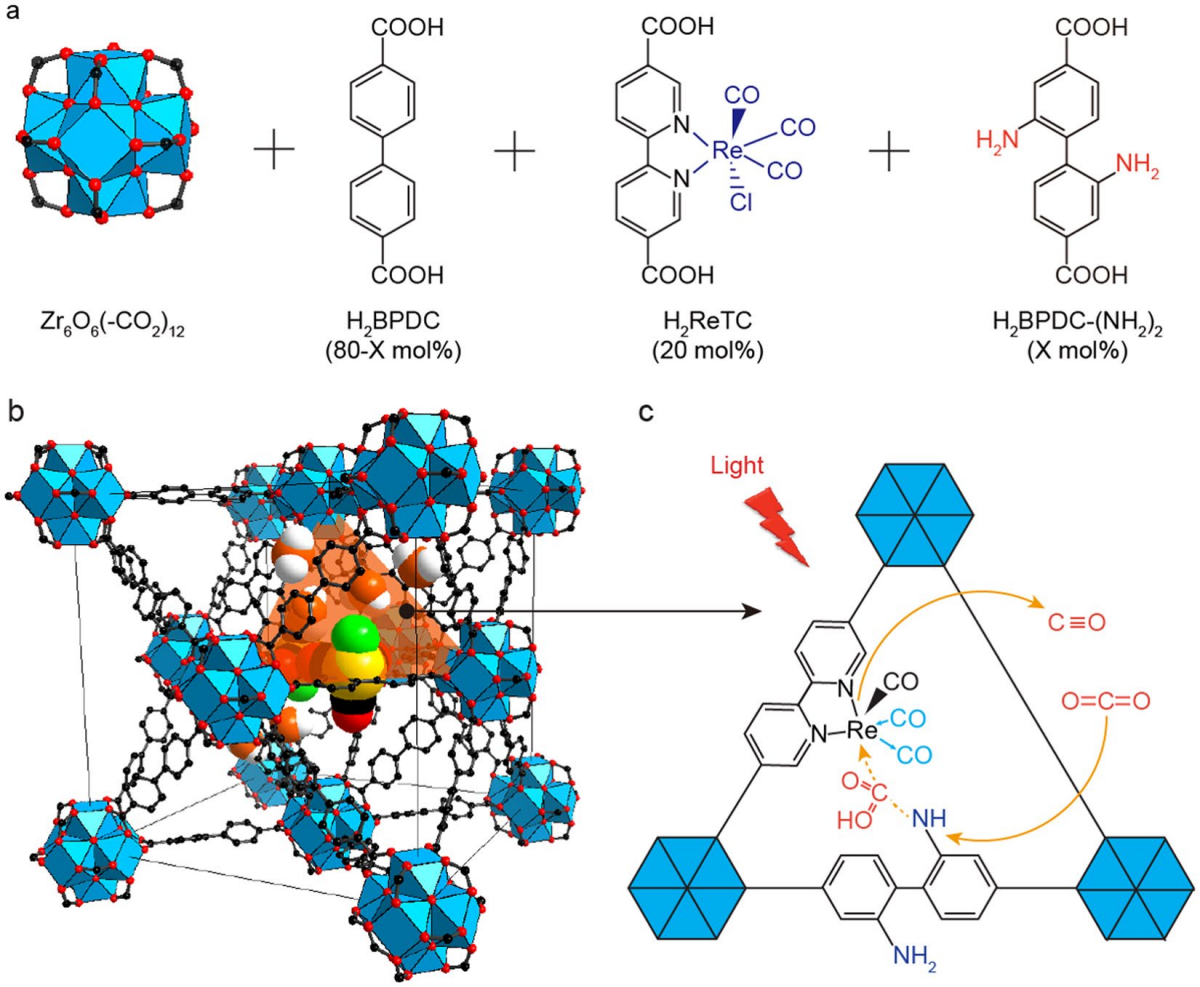
Schematic diagram for the ─NH_2_-functionalized Re-MOF that converts CO_2_ to CO under visible light. (**a**,**b**) Zr_6_O_4_(OH)_4_(–CO_2_)_12_ secondary building units (SBUs) are combined with H_2_BPDC, H_2_ReTC and H_2_BPDC-(NH_2_)_2_ linkers to form Re-MOF-(NH_2_)(X%). The structure of Re-MOF-NH_2_ (X%) is shown. Twelve-coordinated Zr-based metal clusters are interconnected by BPDC, ReTC and BPDC-(NH_2_)_2_ linkers in a face-centred cubic array. Atom labelling scheme: C, black; O, red; Zr, blue polyhedra; Re, yellow; Cl, green; H atoms are omitted for clarity. (**c**) A schematic diagram of the photocatalytic CO_2_ conversion within Re-MOF-NH_2_ (X%).

Herein, we report a new MOF with multiple functional ligands enabling structural conjugation for photocatalytic conversion of CO_2_, where a fine balance in the proximity of two functional groups inside the molecular MOF catalyst is proven to result in cooperative photocatalytic activity for carbon dioxide conversion under visible light. We chose the amine (-NH_2_) functional group as the chemical functionality for incorporation into Re-MOF^9–13^ with ReI(CO)_3_(BPYDC)(Cl), BPYDC = 2,2′-bipyridine-5,5′-dicarboxylate, hereafter referred to as ReTC, which is covalently attached within a zirconium MOF based on a UiO-67-type structure^14^. The -NH_2_ functionalized Re-MOF having a controlled amount of -NH_2_ groups in mol% of X to the total amount of organic linkers is also denoted as Re-MOF-NH_2_ (X%) and Fig. 1 shows that the -NH_2_ functional groups contribute to make the molecular structure of ReTC asymmetric while it helps to form the CO_2_ intermediate within Re-MOF-NH_2_ (33%). MOFs are doped with^9–13^, built by one kind^15–21^ of molecular ligands, and/or combined with inorganic nanomaterials^9, 12, 13, 22^ to give varying levels of photocatalytic activities. Our work has been concentrated on realizing the molecular photocatalysts capable of giving unique cooperative functionality through structural conjugation of two different ligand centres within MOFs. Moreover, the combined experimental methods using inductively coupled plasma atomic emission spectroscopy (ICP-AES) analysis, X-ray diffraction (XRD) patterns, extended X-ray absorption fine structure (EXAFS) spectra, ultraviolet-visible (UV-vis) and *in*-*situ* infrared (IR) spectra, X-ray photoelectron spectroscopy (XPS) scanning electron microscope (SEM) and tunnelling electron microscope (TEM) images, and gas chromatograph data have been also used to probe the cooperative photocatalytic conversion mechanism of carbon dioxide on Re-MOF-NH_2_ having the conjugated ReTC and amine functionalized ligand centres.

## Results and Discussion

### Sample preparation

**Figure 2:**
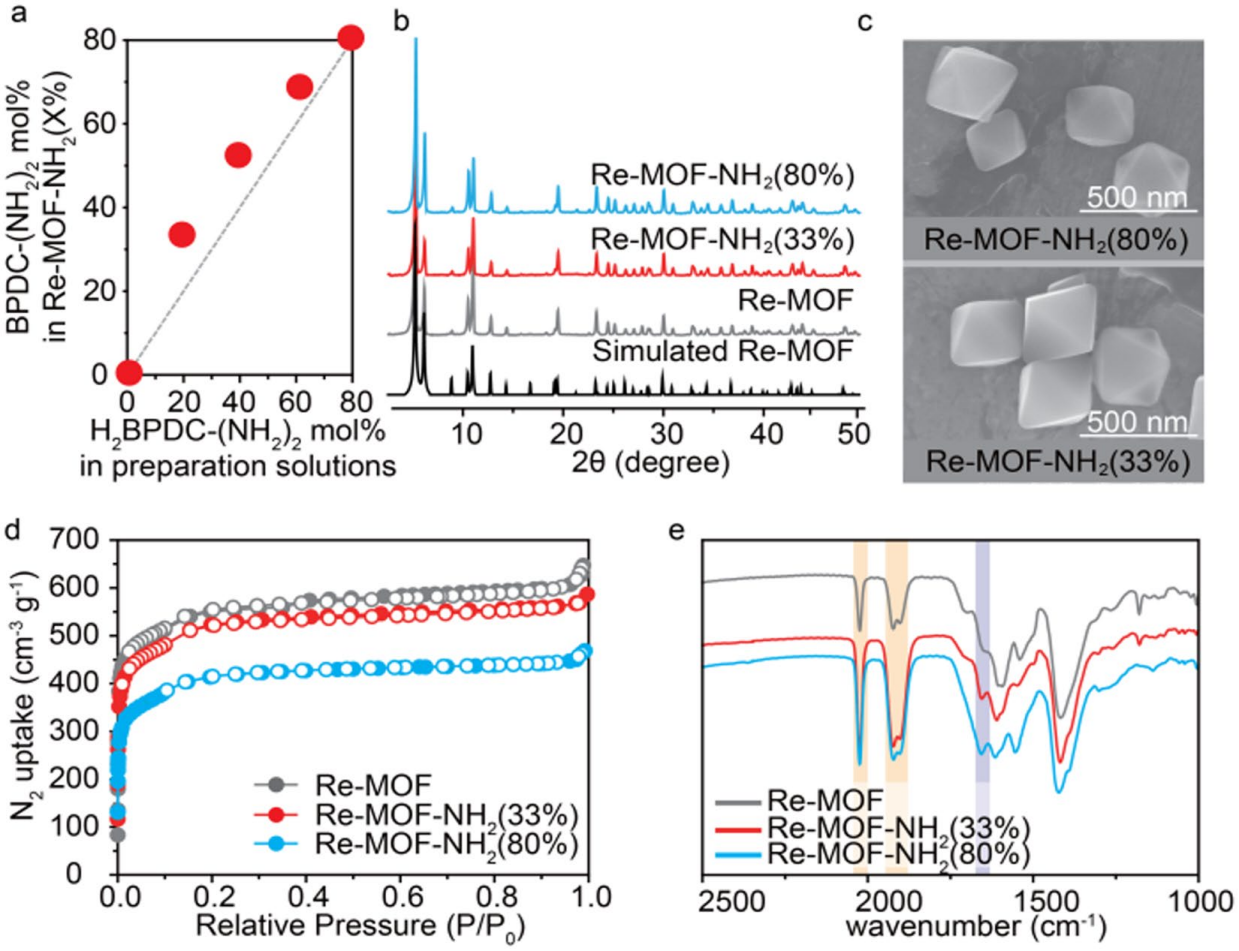
Structural analysis of Re-MOF-NH_2_ (X%). (**a**) Percent incorporation of BPDC-(HN_2_)_2_ in Re-MOF-NH_2_ (X%). (**b**) PXRD patterns of Re-MOF, Re-MOF-NH_2_ (33%) and Re-MOF-NH_2_ (80%) in comparison with the simulated pattern of Re-MOF. (**c**) SEM images of Re-MOF-NH_2_ (33%) and Re-MOF-NH_2_ (80%). (**d**) N_2_ adsorption isotherms for Re-MOF, Re-MOF-NH_2_ (33%) and Re-MOF-NH_2_ (80%) at 77 K with adsorption and desorption points represented by closed circles and open circles, respectively (*P*/*P*_*0*_, relative pressure). (**e**) IR spectra of Re-MOF, Re-MOF-NH_2_ (33%) and Re-MOF-NH_2_ (80%).

The Re-MOF-NH_2_ (X%) samples were prepared by varying the ratio of 2,2′-diaminobiphenyl-4,4′-dicarboxylic acid [H_2_BPDC-(NH_2_)_2_, Fig. S1 in the Supplementary Information] in the range of 0 mol% (-NH_2_-free Re-MOF) to 80 mol% (-NH_2_ at the maximal loading), where the experiment was carried out by increasing the amount of H_2_BPDC-(NH_2_)_2_ by 20, 40, 60, and 80 mol% compared to the total amount of H_2_ReTC and 4,4′-biphenyldicarboxylic acid (H_2_BPDC). The optimal amount of H_2_ReTC was determined to be 20 mol% in the preparation solution of Re-MOF-NH_2_ (X%) for CO_2_ conversion. The amount of H_2_ReTC (Fig. 1a) depends on the ratio of H_2_BPDC-(NH_2_)_2_. The protonated combinations of these three linkers and ZrCl_4_ were dissolved in a mixed solution of DMF/acetic acid in a 20-mL screw-capped vial, which were then heated at 85 °C for 12 hours. The orange suspensions produced in these processes were collected and washed three times with DMF using a centrifuge (8000 rpm for 10 minutes) and sonication. Then, it has been sequentially immersed in methanol for three 24-hour periods. Finally, the samples were activated by removing the solvent under vacuum for 12 hours at room temperature. These processes were found to result in Re-MOF-NH_2_ (X%) with X = 0, 33, 52, 68, and 80, which were identified (Fig. 2a) by the combined experiments using the inductively coupled plasma atomic emission spectroscopy (ICP-AES) and the ^1^H NMR spectroscopy of a digested solution of these samples (Fig. S2 and Table S1 in the Supplementary Information).

### Structural characterization of Re-MOF-NH_2_ (X%)

The crystallinity of Re-MOF-NH_2_ (X%) was determined by the PXRD analysis (Figs 2b and S3 in the Supplementary Information), which gave sharp diffraction lines matching with those of the simulated pattern obtained from the experimental X-ray crystal diffraction data of Re-MOF. This gives a clear evidence for the preservation of the Re-MOF structural arrangement upon introduction of -NH_2_ functional groups at varied ratios and it is notable that all the samples were synthesized as nanoparticle morphologies. The representative scanning electron microscopy (SEM) and transmission electron microscopy (TEM) images (Figs 2c and S4 in the Supplementary Information) of Re-MOF-NH_2_ (X%) demonstrate further that the size of the particles is approximately 300 nm and particles in all the samples have octahedral geometries, regardless of the amount of -NH_2_ functional groups incorporated. The permanent porosity of all the samples was found to be preserved, as confirmed by measurement of the N_2_ adsorption isotherms (Figs 2d and S5 in the Supplementary Information). The results showed Type I isotherms similar to those of Re-MOFs and UiO-67^9, 14^. The Langmuir surface areas for Re-MOF, Re-MOF-NH_2_ (33%), Re-MOF-NH_2_ (52%), Re-MOF-NH_2_ (68%), and Re-MOF-NH_2_ (80%) were 2049, 1900, 1845, 1641, and 1500 m^2^ g^−1^, respectively. This difference in the surface areas was attributed to the amount of the -NH_2_ functional groups and the cramped pore structure in the presence of mixed functional groups.

### Chemical analysis of Re-MOF-NH_2_ (X%)

The presence of -NH_2_ groups and ReTC in Re-MOF-NH_2_ (X%) was confirmed through IR spectra (Figs 2e and S6 in the Supplementary Information). The peak at 1655 cm^−1^ was assigned to the N-H bending mode of the -NH_2_ functional group, while those at 2022, 1920, and 1910 cm^−1^ correspond to the C≡O stretching ones of ReTC^9, 23^. In addition, the UV-vis spectra and XPS results of Re-MOF-NH_2_ (X%) support the presence of ReTC, BPDC-(NH_2_)_2_, and BPDC (Figs S7 and S8 in the Supplementary Information). The results show that chemical groups of NH_2_ and C≡O remain unchanged during synthesis. The ratios of functional group, determined by the ^1^H NMR spectroscopy of a digested solution (Figs 2a and S2 in the Supplementary Information), were shown to result in Re-MOF, Zr_6_O_4_(OH)_4_(ReTC)_1.62_(BPDC)_4.38_; Re-MOF-NH_2_ (33%), Zr_6_O_4_(OH)_4_(ReTC)_1.62_(BPDC-(NH_2_)_2_)_1.98_(BPDC)_2.43_; Re-MOF-NH_2_ (52%), Zr_6_O_4_(OH)_4_(ReTC)_1.62_(BPDC-(NH_2_)_2_)_3.12_(BPDC)_1.44_; Re-MOF-NH_2_ (68%), Zr_6_O_4_(OH)_4_(ReTC)_1.26_(BPDC-(NH_2_)_2_)_4.08_(BPDC)_0.63_; Re-MOF-NH_2_ (80%), Zr_6_O_4_(OH)_4_(ReTC)_1.20_(BPDC-(NH_2_)_2_)_4.8_.

### Photocatalytic activity and stability of Re-MOF-NH_2_ (X%)

**Figure 3:**
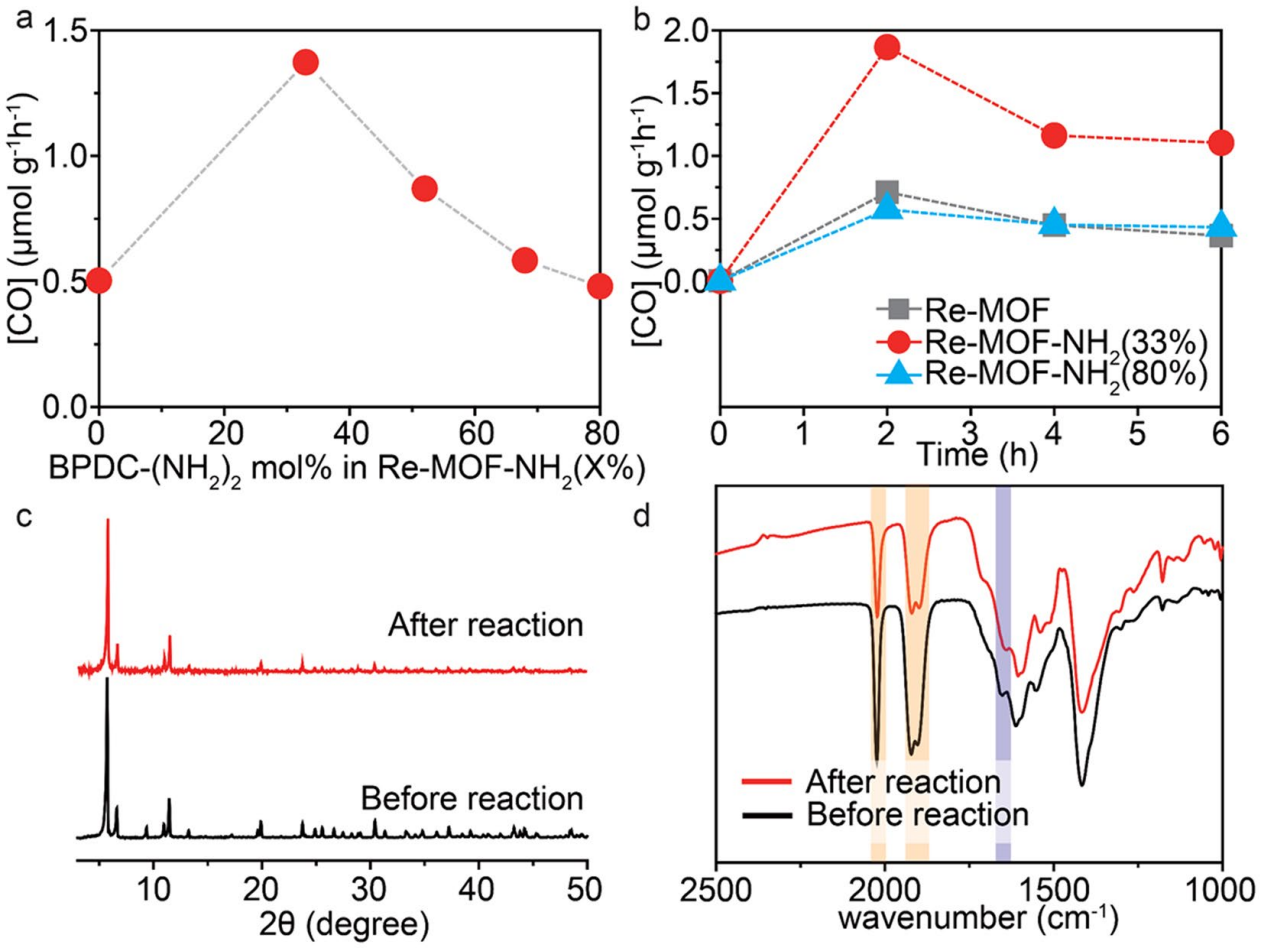
Photocatalytic activity and stability of Re-MOF-NH_2_ (X%). (**a**) Photocatalytic CO_2_-to-CO conversion activity under visible light (400–700 nm). (**b**) Photocatalytic CO_2_ conversion rate under visible light. (**c**) PXRD of Re-MOF-NH_2_ (33%) before and after the reaction. (**d**) IR spectra of Re-MOF-NH_2_ before and after the reaction.

All Re-MOF-NH_2_ (X%) samples were explored for photocatalytic CO_2_ conversion rate under visible light. The samples, which were kept in vacuum overnight, were placed in a stainless-steel reactor and purged with CO_2_ and the conversion was initiated in the presence of triethylamine (TEA) under visible light (400–700 nm) using a 300 W Xenon arc lamp. The products produced through the photocatalytic reaction were analysed and quantified using the gas chromatography (GC) equipped with a flame ionization detector (FID) and normalized to the mass of the sample. Figure 3a shows photocatalytic activities of the Re-MOF-NH_2_ (X%) samples. The results show that the photocatalytic conversion is the highest when 33 mol% of -NH_2_ functional groups, denoted as Re-MOF-NH_2_ (33%), is incorporated. Moreover, its activity is proven to be 3-folds higher than that of Re-MOF with no -NH_2_ groups (Fig. 3a). In the absence of CO_2_ under a He atmosphere or with no ReTC, no CO generation was observed. The high activity was maintained for at least 6 hours while the molecular catalyst of H_2_ReTC was found to be deactivated within 1 hour^9, 24^, attributed to both ReTC and -NH_2_ functional groups covalently bound to the Re-MOF-NH_2_ (X%) framework. This signals that it prevents the prevailing deactivation pathway of dimerization commonly observed in photoactive molecular complexes. The stability of the Re-MOF-NH_2_ (33%) sample was also tested after the photocatalytic reaction. The PXRD, SEM and TEM analyses (Figs 3c and S9 in the Supplementary Information) show that their crystallinity and morphology are well maintained after the photocatalytic reaction. In addition, the IR spectra of Re-MOF-NH_2_ (33%) before and after the reaction (Fig. 3d) demonstrate that conjugation of ReTC and -NH_2_ functional groups preserves their chemical configurations after photocatalysis. This is a clear evidence that Re-MOF-NH_2_ (33%) enables to give a high conversion rate with the 100% selectivity for conversion of CO_2_ into CO under visible light while maintaining its crystal structure, morphology and chemical functionality after the photocatalytic reaction.

### EXAFS analysis to determine the cooperative mechanism for photocatalytic conversion

**Figure 4:**
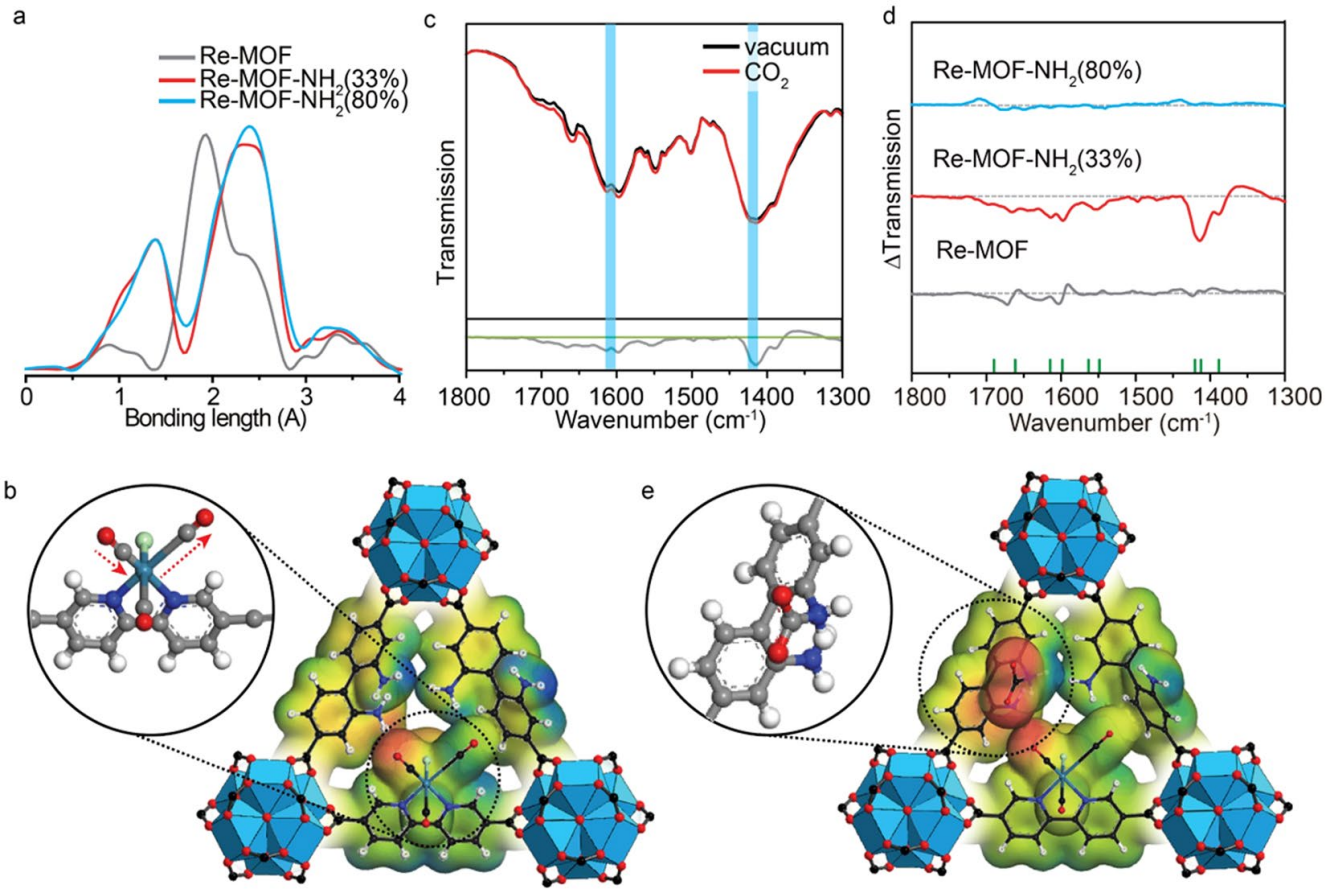
Characterization of the photocatalytic conversion mechanism in Re-MOF-NH_2_ (33%). (**a**) EXAFS spectra for Re-MOF, Re-MOF-NH_2_ (33%) and Re-MOF-NH_2_ (80%). (**b**) Electrostatic potential diagram for Re-MOF-NH_2_ (33%) where the ReTC inside is asymmetric. (**c**) IR spectra of Re-MOF-NH_2_ (33%) under vacuum and CO_2_ atmosphere. (**d**) Difference IR spectra of Re-MOF, Re-MOF-NH_2_ (33%) and Re-MOF-NH_2_ (80%) in the presence of CO_2_. (**e**) Electrostatic potential diagram of the intermolecular stabilization of carbamate by the -NH_2_ functional groups before making CO in Re-MOF-NH_2_ (33%).

The photocatalytic activity in Re-MOF-NH_2_ (33%) should be related to the interaction between the ReTC and its neighbouring -NH_2_ functional group. The EXAFS analysis, which was performed for Re-MOF, Re-MOF-NH_2_ (33%), and Re-MOF-NH_2_ (80%) at the Pohang Accelerator Laboratory (PAL, Republic of Korea) on the 7D beamline at 3 GeV energy and the Re L_3_-edge spectra (E_0_ = 10207 eV), were also performed in the transmission mode^25, 26^ to measure the bonding length between the Re metal complex centre and the neighbouring atoms (Fig. 4a). The spectra were obtained through the Athena software based on the IFEFFIT library. The results demonstrate that Re-MOF without any -NH_2_ functional group gives two bonding lengths of 1.8 Å and 2.3 Å, corresponding to the bonds for Re-C and Re-Cl in ReTC, respectively. When the -NH_2_ functional groups are introduced into Re-MOF-NH_2_ (33%) and Re-MOF-NH_2_ (80%), we found that the bond corresponding to Re–C was divided into two bonding lengths of 1.4 Å and 2.3 Å. This is because the configuration of carbonyl groups in ReTC becomes asymmetric upon the interaction with the -NH_2_ functional groups. Figure 4b gives the clear evidence that the distance between the Re centre and each carbonyl group becomes different. This asymmetric configuration of the carbonyl groups leads to the uneven electron distribution in ReTC for Re-MOF-NH_2_ (X%), which would contribute to polarization of ReTC and thus increase the partial electron affinity to make better chance converting CO_2_ to CO when carbamate is formed upon interaction with the -NH_2_ functional groups.

### IR spectroscopy of Re-MOF-NH_2_ (X%) in the presence of CO_2_

The results via the EXAFS analysis could not explain why Re-MOF-NH_2_ (33%) gives a higher activity than Re-MOF-NH_2_ (80%). In this reason, we have investigated further the interaction between the -NH_2_ functional group and CO_2_ using the *in*-*situ* IR spectroscopy, where each sample of Re-MOF, Re-MOF-NH_2_ (33%), and Re-MOF-NH_2_ (80%) was kept inside the chamber and the IR spectra were measured with the chamber under vacuum or filled with CO_2_. The IR spectra of Re-MOF-NH_2_ (33%) are shown in Fig. 4c and those of Re-MOF and Re-MOF-NH_2_ (80%) are shown in Fig. S10a,b in the Supplementary Information. Figure 4d also show the difference spectra of each sample in the presence of CO_2_. The results give that differences in the spectra are evident only in Re-MOF-NH_2_ (33%) and that the most pronounced peaks are at 1596 and 1420 cm^−1^ corresponding to the asymmetric and symmetric COO- stretching vibrations, respectively^27^. This shows that amine functional groups helped to result in the intermolecular stabilization of carbamate as shown in Fig. 4e.

### Theoretical investigation for photocatalytic cooperative conversion mechanism of CO_2_ into CO

**Figure 5:**
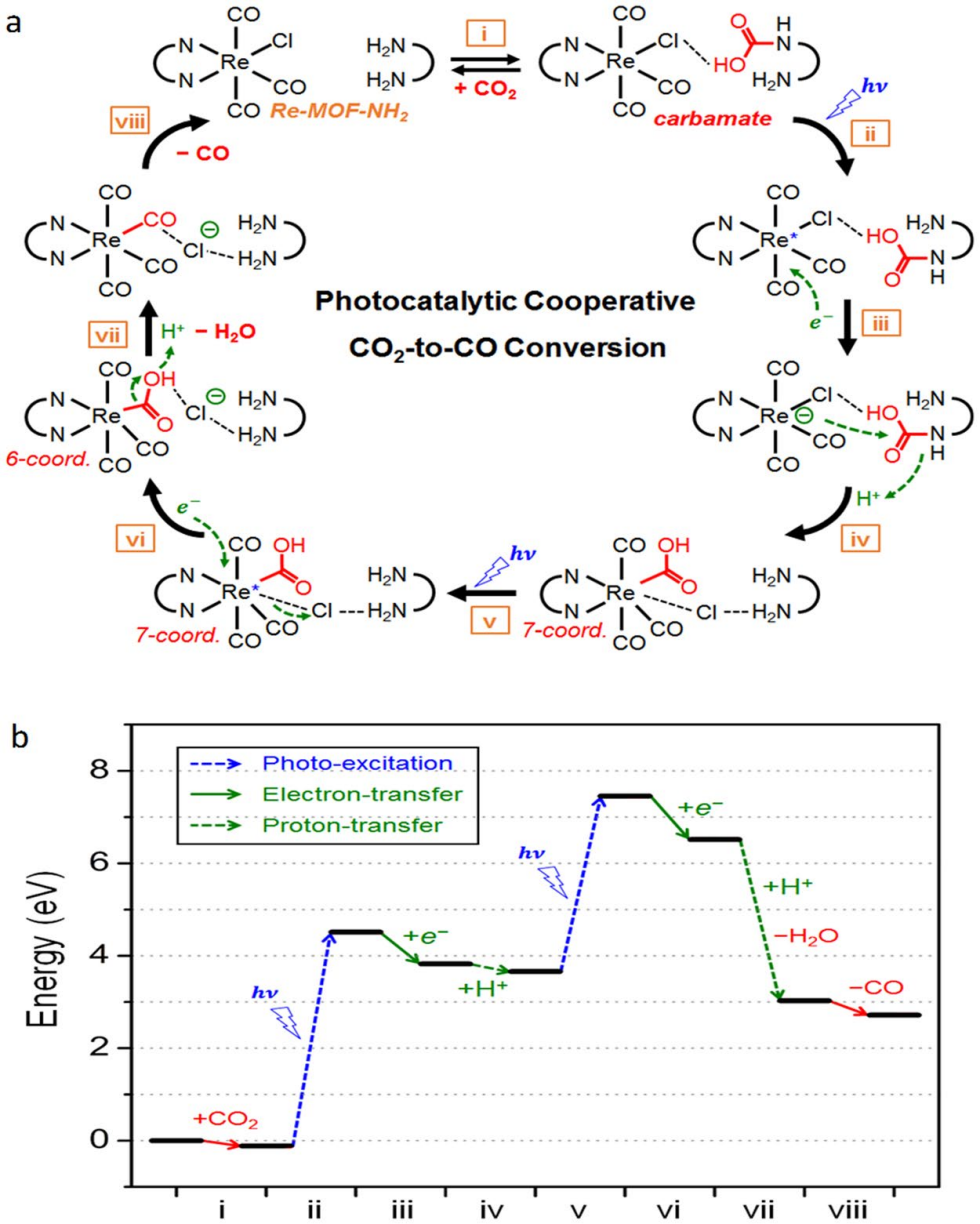
Proposed amine-assisted synergetic photocatalytic conversion mechanism of CO_2_ into CO based on DFT energetics. (**a**) The reaction paths for photocatalytic conversion of CO_2_ into CO; (i) the carbamate formation, (ii) the 1^st^ photo-excitation, (iii) the 1^st^ electron transfer from TEA, (iv) the nucleophilic attack of Re to COOH accompanied with the 1^st^ proton transfer from TEA^+^, (v) the 2^nd^ photo-excitation, (vi) the 2^nd^ electron transfer from TEA, (vii) the H_2_O formation accompanied with the 2^nd^ proton transfer from TEA^+^, and (viii) the CO formation. (**b**) The calculated energies from stage i to stage viii. The results demonstrate that the production of CO upon absorption of two photons in visible light is energetically favorable in the presence of the –NH_2_ ligand and the ReTC ligand in the neighboring sites.

Figure 5a shows the stable CO_2_–to–CO photochemical reduction pathway determined by using density functional theory (DFT) calculations. The first reaction corresponding to step i occurs via formation of the carbamate complex that is initiated by adsorption of CO_2_ into the -NH_2_ ligand, where the OH of the carboxylic group forms a week hydrogen bonding interaction with the Cl attached in a neighboring ReTC ligand (Fig. S11a,b). Steps ii and iii in Fig. 5a give clear evidences that the electron uptake from TEA spontaneously occurs after the photo-excitation process. Due to the additional electron injected in the ReTC, the Re center attacks the electrophilic carbonyl carbon of a nearby –COOH accompanying with the protonation of amine group where the proton is from TEA^+^. This leads to the migration of the –COOH group to the ReTC ligand along with the regeneration of -NH_2_ group (step iv). It is notable that the Re center then forms a 7-coordinate system (Fig. S11c). Subsequently, upon the second photo-excitation process (step v) followed by the second electron and proton uptake from TEA (step vi and vii), the –COOH group is transformed to the final product of CO and the byproduct of H_2_O at the ReTC ligand. Consequently, these support that the formation of the carbamate intermediate in the presence of the -NH_2_ ligand is responsible to give efficient production of CO from CO_2_ in Re-MOF-NH_2_ via the synergistic interaction of two different ReTC and -NH_2_ ligands that is not available in Re-MOF having no adjacent amine moiety. Furthermore, the corresponding energy diagram from step i to viii (Fig. 5b) gives more clarifications that the production of CO upon absorption of two photons in visible light is energetically favorable in the presence of the -NH_2_ ligand and the ReTC ligand in the neighboring sites. It is also notable that the structural details involving in the photocatalytic reaction have been summarized in Fig. S12.

On summary, we designed the chemical environment of a molecular photocatalyst covalently bound to the MOFs through incorporation of the -NH_2_ functionalized ligand resulting in a conjugated structure of the neighbouring Re metal complex ligand. The ratio of the -NH_2_ functional groups was varied from 0 to 80 mol% in Re-MOF, and the results showed that the photocatalytic CO_2_ to CO conversion reached the highest activity when 33 mol% of the -NH_2_ functional groups was incorporated. In addition, the EXAFS and IR spectra supported the cooperative reaction mechanism in Re-MOF-NH_2_ (33%). These results revealed that the -NH_2_ functional groups induced the different bond lengths for Re-CO in ReTC while they help to make the intermolecular stabilization of carbamate with CO_2_, thus increasing its photocatalytic activity.

## Methods

### Synthesis of Re-MOF-NH_2_ (X%)

We have prepared the ligand and metal units for the MOF separately. First, ZrCl_4_ (9.3 mg, 0.04 mmol) was dissolved in N,N’-dimethylformamide (DMF, 5 mL) with acetic acid (0.5 mL) in a 20 mL glass vial. The ligand was prepared using a solution of rhenium ligands (4.4 mg, 0.008 mmol), H_2_-BPDC-(NH_2_)_2_ (0, 20, 40, 60, and 80 mol% of H_2_ReTC), biphenyl-4,4′-dicarboxylic acid (adding up to a total amount of 0.040 mmol of organic linker) and DMF (5 mL). Then, the well-dispersed ligand solution was poured into the metal solution, and the sealed vial was placed in an 85 °C oven for 12 hours without light. Then, it was cooled to room temperature and the solid was separated with a centrifuge (8000 rpm for 10 minutes). Next, it was washed with DMF once and methanol three times. After washing, the product was dried in a vacuum oven.

### Photocatalytic measurements

The photocatalytic CO_2_ conversion experiments were conducted using a stainless-steel reactor. To accomplish this, 5 mg of the sample was placed on a glass holder and the reactor was fastened with screws. To remove solvent and air, the reactor was kept under vacuum overnight. Then, the reactor was purged using CO_2_ gas (99.9%) for 30 minutes. Next, 0.1 mL of trimethylamine (TEA) was injected into the chamber, which was kept at 40 °C for approximately 15 minutes to vaporize the TEA. The sample was irradiated through the quartz glass using a 300 W Xenon arc lamp (ORIEL) equipped with an IR water filter and a UV filter to obtain visible light ranging from 400 to 700 nm. The evolved gas was sampled using an airtight sample-lock syringe (Hamilton, 81256) and was injected into a gas chromatography column (Shimadzu, GC-2014A) and passed through a HayeSep Q Porapak Q column with helium carrier gas. The gas was detected using a flame ionization detector (FID) and a methanizer.

### EXAFS analysis

The extended X-ray absorption fine structure (EXAFS) analysis was conducted at the Pohang Accelerator Laboratory (PAL) on the 7D beamline at 3 GeV energy. A Si(111) double crystal monochromator was used to monochromatize the synchrotron radiation and the Re L3-edge spectra (E_0_ = 10207 eV) were obtained in transmission mode. To minimize contamination of higher harmonics, the incident beam was detuned by 10%. Also, the intensity of the beam was monitored by using a N_2_-filled IC SPEC ionization chamber. This experiment was conducted with 0.4 eV steps between 10515 and 10585 eV, 30 eV steps between 10585 and 11707 eV, and 50 eV steps between 11707 and 12007 eV with 2, 3, and 3 seconds per the point, respectively. Then, the spectra were obtained through the Athena software based on the IFEFFIT library.

### *In*-*situ* IR Spectroscopy

The *in*-*situ* IR spectroscopy was measured by the FT-IR spectrometer equipped with a gas chamber. The Re-MOF-NH_2_ (X%) samples were diluted with the KBr powder and placed in the sample holder. Then, before measuring the IR spectrum in vacuum, the chamber was evacuated for 30 minutes. Next, the chamber was filled with CO_2_ gas for 30 minutes to measure the IR signal under CO_2_ atmosphere. Each sample was measured with a 4 cm^−1^ spectral resolution and a 4 mm/cm scanning speed.

#### Computational Details

DFT calculations were performed using the Jaguar 8.9 software^28^ for theoretical reaction energetics. We used the range-separated exchange-correlation functional of CAM-B3LYP^29^ the reliable description of band edge positions, and used the LACVP basis set^30^. Also, the ground electronic and geometric structures were fully optimized under constraints with the fixed coordinates of oxygen atoms in terminal carboxylic functional groups from experimentally observed atomic positions. For the steps involving electron/proton transfer, the electron transfer is determined on consideration of coupling with the oxidation of TEA corresponding to the reaction of TEA → TEA^+^ + −e^−^ while the proton is transferred from the alpha carbon of TEA^+^. The photo-excitation energy is calculated using the HOMO-LUMO gap.

## Electronic supplementary material

Supporting information

## References

[1] Gade, L. H. & Hofmann, P. *Molecular Catalysts*: *Structure and Functional Design* (Wiley, 2014).

[2] Okada, Tatsuhiro & Kaneko, Masao. *Molecular Catalysts for Energy Conversion* (Springer, 2009).

[3] Blakemore JD, Crabtree RH, Brudvig GW (2015). Molecular Catalysts for Water Oxidation. Chem. Rev..

[4] White JL (2015). Light-Driven Heterogeneous Reduction of Carbon Dioxide: Photocatalysts and Photoelectrodes. Chem. Rev..

[5] Berardi S (2014). Molecular Artificial Photosynthesis. Chem. Soc. Rev..

[6] Aresta M, Dibenedetto A, Angelini A (2013). Catalysis for the Valorization of Exhaust Carbon: from CO_2_ to Chemicals, Materials, and Fuels. Technological Use of CO_2_. Chem Rev..

[7] Schulz H (1999). Short History and Present Trends of Fischer–Tropsch Synthesis. App Catal A: General..

[8] Centi, G., Quadrelli, E. A. & Perathoner, S. Catalysis for CO_2_ Conversion: A Key Technology for Rapid Introduction of Renewable Energy in the Value Chain of Chemical Industries. *Energy & Environ Sci*. **6**, pp. 1711–1731 (2013).

[9] Choi, K. *et al*. Plasmon-Enhanced Photocatalytic CO_2_ Conversion within Metal-Organic Frameworks Under Visible Light. *J*. *Am*. *Chem*. *Soc*., doi:10.1021/jacs.6b11027. 10.1021/jacs.6b11027 28004911

[10] Wang C, Xie Z, deKrafft KE, Lin W (2011). Doping Metal-Organic Frameworks for Water Oxidation, Carbon Dioxide Reduction, and Organic Photocatalysis. J. Am. Chem. Soc..

[11] Blake AJ (2010). Photoreactivity Examined through Incorporation in Metal-Organic Frameworks. Nat. Chem..

[12] Zhang T, Lin W (2014). Metal–Organic Frameworks for Artificial Photosynthesis and Photocatalysis. Chem. Soc. Rev..

[13] Wang J, Wang C, Lin W (2012). Metal–Organic Frameworks for Light Harvesting and Photocatalysis. ACS Catalysis.

[14] Cavka JH (2008). A New Zirconium Inorganic Building Brick Forming Metal Organic Frameworks with Exceptional Stability. J. Am. Chem. Soc..

[15] Lee Y, Kim S, Kang JK, Cohen SM (2015). Photocatalytic CO_2_ Reduction by a Mixed Metal (Zr/Ti), Mixed Ligand Metal–Organic Framework under Visible Light Irradiation. Chem. Commun..

[16] Fei H, Sampson MD, Lee Y, Kubiak CP, Cohen SM (2015). Photocatalytic CO_2_ Reduction to Formate Using a Mn(I) Molecular Catalyst in a Robust Metal-Organic Framework. Inorg. Chem..

[17] Fu Y (2012). An Amine-Functionalized Titanium Metal-Organic Framework Photocatalyst with Visible-Light-Induced Activity for CO_2_ Reduction. Angew. Chem. Int. Ed..

[18] Wang D, Huang R, Liu W, Sun D, Li Z (2014). Fe-Based MOFs for Photocatalytic CO_2_ Reduction: Role of Coordination Unsaturated Sites and Dual Excitation Pathways. ACS Catal..

[19] Wang S, Yao W, Lin J, Ding Z, Wang X (2014). Cobalt Imidazolate Metal-Organic Frameworks Photosplit CO_2_ under Mild Reaction Conditions. Angew. Chem. Int. Ed..

[20] Easun TL (2014). Photochemistry in a 3D Metal-Organic Framework (MOF): Monitoring Intermediates and Reactivity of the Fac-to-Mer Photoisomerization of Re(diimine)(CO)_3_Cl Incorporated in a MOF. Inorg. Chem..

[21] Hod I (2015). Fe-Porphyrin Based MOF Films as High-Surface-Concentration, Heterogeneous Catalysts for Electrochemical Reduction of CO_2_. ACS Catal..

[22] Wang C, Krafft KE, Lin W (2012). Pt Nanoparticles@Photoactive Metal–Organic Frameworks: Efficient Hydrogen Evolution via Synergistic Photoexcitation and Electron Injection. J. Am. Chem. Soc..

[23] Zhao HC (2013). Investigation of Monomeric versus Dimeric fac-Rhenium(I) Tricarbonyl Systems Containing the Noninnocent 8-Oxyquinolate Ligand. Organometallics.

[24] Benson EE, Kubiak CP (2012). Structural Investigations into the Deactivation Pathway of the CO_2_ Reduction Electrocatalyst Re(bpy)(CO)_3_Cl. Chem. Commun..

[25] Surblé S, Millange F, Serre C, Férey G, Walton RI (2006). An EXAFS Study of the Formation of a Nanoporous Metal-Organic Framework: Evidence for the Retention of Secondary Building Units during Synthesis. Chem. Commun..

[26] Macnaughtan ML, Soo HS, Frei H (2014). Binuclear ZrOCo Metal-to-Metal Charge-Transfer Unit in Mesoporous Silica for Light-Driven CO_2_ Reduction to CO and Formate. J. Phys. Chem. C.

[27] Hahn MW, Jelic J, Berger E, Reuter K, Jentys A, Lercher JA (2016). Role of Amine Functionality for CO_2_ Chemisorption on Silica. J. Phys. Chem. B.

[28] Bochevarov AD (2013). Jaguar: A High-Performance Quantum Chemistry Software Program with Strengths in Life and Materials Sciences. Int. J. Quantum Chem..

[29] Yanai T, Tew DP, Handy NC (2004). A New Hybrid Exchange–Correlation Functional Using the Coulomb-Attenuating Method (CAM-B3LYP). Chem. Phys. Lett..

[30] Hay PJ, Wadt WR (1985). Ab Initio Effective Core Potentials for Molecular Calculations: Potentials for K to Au Including the Outermost Core Orbitals. J. Chem. Phys..

