# Supplementary material for: Synergistic interaction of Re complex and amine functionalized multiple ligands in metal-organic frameworks for conversion of carbon dioxide
Source: Sci Rep. 2017 Apr 4;7:612. doi: 10.1038/s41598-017-00574-1 (PMC5428566; doi:10.1038/s41598-017-00574-1)
Supplement: Supplementary file 1 — Supporting information [file 41598_2017_574_MOESM1_ESM.doc]

Supporting Information for

**Synergistic interaction of Re complex and amine functionalized multiple ligands in metal-organic frameworks for conversion of carbon dioxide**

*Un Jin Ryu^1,2‡^, Sang Jun Kim^3‡^, Hyung-Kyu Lim^1‡^, Hyungjun Kim^1*^, Kyung Min Choi^2*^ and Jeung Ku Kang^1,3*^*

^1^Graduate School of Energy, Environmental, Water and Sustainability (EEWS), Korea Advanced Institute of Science and Technology, 291 Daehak-ro, Yuseong-gu, Daejeon 34141, Republic of Korea

^2^Department of Chemical and Biological Engineering, Sookmyung Women’s University, 100 Cheongpa-ro 47 gil, Yongsan-gu, Seoul 04310, Republic of Korea

^3^Department of Materials Science and Engineering, Korea Advanced Institute of Science and Technology, 291 Daehak-ro, Yuseong-gu, Daejeon 34141, Republic of Korea

^‡^Contributed equally to this work.

*Corresponding authors: [linus16@kaist.ac.kr](mailto:linus16@kaist.ac.kr), [kmchoi@sookmyung.ac.kr](mailto:kmchoi@sookmyung.ac.kr), jeung@kaist.ac.kr

**Table of Contents**

**Section S1.** Materials and methods

**Section S2.** Chemical analysis of H_2_BPDC-(NH_2_)_2_

**Section S3.** Characterizations of Re-MOFs

**Section S4.** References

**Section S1.** Materials and methods

**S1.1. Experimental details**

***Chemicals***

Rhenium pentacarbonyl chloride (98%, Acros), 2,2'–bipyridine-5,5'-dicarboxylic acid (98.0%, TCI), biphenyl-4,4’-dicarboxylic acid (97%, Aldrich), Zirconium (IV) chloride (≥99.5% trace metals basis, Aldrich), N,N-Dimethyl formamide (Guaranteed Reagent, JUNSEI), acetic acid (≥99.7%, Aldrich), methyl-4-iodo-3-nitrobenzoate (99%, Alfa Aesar), copper powder (<425 micron, 99.5% metals, Sigma-Aldrich), Na_2_S_2_O_3_·5H_2_O (≥99.5%, Aldrich), iron fine powder (≥99%, Aldrich), MgSO_4_ (Sigma), methanol (anhydrous, Sigma-Aldrich), THF (anhydrous, Sigma-Aldrich), LiOH·H_2_O (Sigma-Aldrich).

***Synthesis of Tris-carbonyl-chloro(5,5'-dicarboxyl-2,2‘-bipyridine) rhenium(I) (H_2_ReTC)*** ^S1^

The tris-carbonyl-chloro(5,5’-dicarboxyl-2,2’-bipyridine) rhenium (I) as rhenium ligand is synthesized by rhenium pentacarbonyl chloride (2.5 mmol, 0.92 g) and 2,2'-bipyridine-5,5'-dicarboxylic acid (0.5 mmol, 0.61 g).1The mixture of these chemicals dissolve in methanol (250 mL) with 500 mL flask. This solution was refluxed at 66ºC under argon atmosphere overnight. During the reaction, the color changed white to orange. The resultant was filtered and the solvent was removed by a rotary evaporator. The product was dried under vacuum at 60ºC to give deep orange powders with the yield of 90% and the ^1^H NMR (DMSO-*d_6_*) of 9.34(s, 2H), 9.00(d, 2H), and 8.77(d, 2H).

***Synthesis of 2,2’-Diaminobiphenyl-4,4’-dicarboxylic acid (H_2_BPDC-(NH­_2_)_2_)*** ^S2^


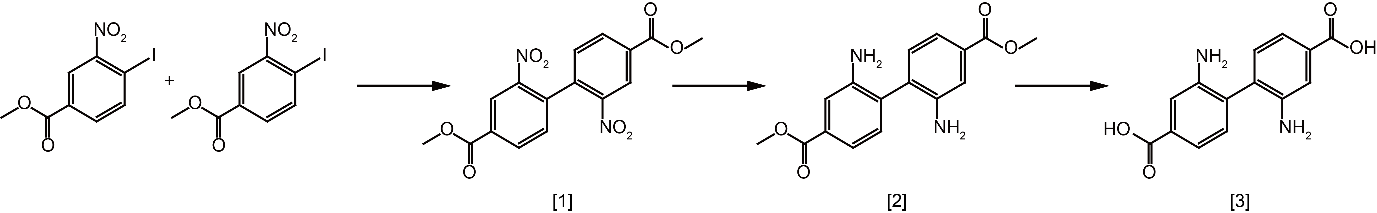


***Synthesis of Dimethyl-2,2’-dinitrobiphenyl-4,4’-dicarboxylate [1]***

The solution of methyl-4-iodo-3-nitrobenzoate (10 g, 32.6 mmol) and copper powder (8.28 g, 130 mmol) in DMF (100 mL) was refluxed under argon atmosphere overnight. After cooled down to room temperature, the resultant was filtered with DMF and quenched with 400 mL 5% Na_2_S_2_O_3_·5H_2_O aqueous solution. The solution was added ethyl acetate and extracted organic layer was washed with brine and dried over anhydrous MgSO_4_. After being filtered and evaporated, product was dried under vacuum to give bright yellow powders with the yield of 90% and the ^1^H NMR (DMSO-*d_6_*) of 8.70(s, 2H), 8.40(d, 2H), 7.73(d, 2H), and 3.96(s, 6H).

***Synthesis of Dimethyl-2,2’-diaminobiphenyl-4,4’-dicarboxylate [2]***

Dimethyl-2,2’-dinitrobiphenyl-4,4’-dicarboxylate (5 g, 13.9 mmol) was dissolved in acetic acid (150 mL) and added iron powder in the flask. The solution was stirred overnight at room temperature. The resultant was filtered over celite and evaporated. The residue was dissolved in ethyl acetate and extracted organic layer was washed with conc. NaHCO_3_, brine and dried over anhydrous MgSO_4_. After being filtered and evaporated, product was dried under vacuum to give yellow powders with the yield of 65% and the ^1^H NMR (DMSO-*d_6_*) of 7.44(s, 2H), 8.24(d, 2H), 7.09(d, 2H), 4.99(s, 4H), and 3.83(s, 6H).

***Synthesis of 2,2’-Diaminobiphenyl-4,4’-dicarboxylic acid [3]***

Dimethyl-2,2’-diaminobiphenyl-4,4’-dicarboxylate (1.6 g, 5.26 mmol) was dissolved in methanol (50 mL) and THF (100 mL). After added 0.6 M LiOH·H_2_O aqueous solution drop by drop, the mixture was stirred overnight at room temperature. The resultant was evaporated to remain water and the residue was dissolved in ethyl acetate and extracted organic layer. The organic solution was concentrated and the residue was acidified with acetic acid. After being filtered and evaporated, the product was dried under vacuum to give bright yellow powders with the yield of 60% and the ^1^H NMR (DMSO-*d_6_*) of 12.65(br, 2H), 7.42(s, 2H), 7.24(d, 2H), 7.07(d, 2H), and 4.94(s, 4H).

***Structural analyses***

The attenuated total reflectance (ATR) FTIR spectra of neat samples were obtained on a JASCO FT/IR-6100FV spectrometer equipped with the ZnSe window. The solution ^1^H nuclear magnetic resonance (NMR) data for ligands were obtained by Bruker AV-300. The ligands sample was dissolved in DMSO-*d_6_* (dimethyl sulfoxide-*d6* (D, 99.9%) + 0.05% v/v TMS, CIL). For the digest-NMR of MOFs, 5 mg of dried sample was digested and dissolved by sonication in a mixture of DMSO-*d_6_* (600 µL), deuterium chloride (200 µL). The digested solution was used directly for ^1^H NMR. The powder x-ray diffraction spectra were obtained by the RIGAKU XRD (Smartlab, Cu-Kα radiation) at 1200W (40 kV, 30 mA). The scanning condition was set up 4°/min scan rate from 3° to 50° with a silicon holder. The morphology and surface of the MOFs were verified by Field Emission Scanning electron microscope (FE-SEM, JEM-7600F, JEOL). The powder sample was dissolved in methanol and dropped on the holder directly. For transmission electron microscopy (TEM) observation, samples were first dispersed in an organic solvent by sonication and dropped onto a TEM grid. TEM was carried out at 200 kV using a JEOL JEM-2100F. The UV-Visible spectrometer (JASCO V-570) was provided light absorption ranges of ligands and MOFs. Samples were detected as powder condition. All scan range was 200-800 nm, and the lamp condition changed from deuterium arc lamp (from 800 nm) exchanged to tungsten lamp at 360 nm. The X-ray photoelectron spectroscopy (XPS, K-alpha, Thermo VG Scientific) was using Al K-alpha radiation with 3 mA of beam current and 400 μm of beam size and number of scan for N was 25. Inductively coupled plasma-optical emission spectrometer (ICP-OES, Agilent ICP-OES 720) was used to detect the components of MOFs. It should be enacted the microwave reaction before detection. Correlation coefficients were 1.000000 (Re) and 0.999999 (Zr), so it shows the data were in confidence range. Gas adsorption analysis was performed on a Quantachrome Instruments Quadrasorb-SI automatic volumetric gas adsorption analyzer. A liquid nitrogen bath (77 K) and ultra-high purity grade N_2_ and He (99.999%, Praxair) were used for the N_2_ adsorption-desorption isotherms. Samples were prepared and measured after evacuating at 100 ºC for 24 h. Specific surface area was calculated by Brunauer-Emmett-Teller(BET) model.

**S1.2. Computational details**

***Density functional calculation (DFT) calculations***
DFT calculations were performed using the Jaguar 8.9 software^S3^ for theoretical reaction energetics. We used the range-separated exchange-correlation functional of CAM-B3LYP^S4^ for the reliable description of band edge positions, and used the LACVP basis set^S5^, where effective core potential is used to describe the heavy elements with reduced computational cost. To avoid computational difficulties, we simplified the Re-MOF-NH_2_ structure as a molecular complex of ReTC and –NH_2_ ligands which are terminated with carboxylic functional groups. The ground electronic and geometric structures were fully optimized under constraints with the fixed coordinates of oxygen atoms in terminal carboxylic functional groups from experimentally observed atomic positions. For the steps involving electron/proton transfer, we assumed that the electron transfer is coupled with the oxidation of TEA (i.e., TEA → TEA^+^ + - e^-^) and the proton is transferred from the alpha carbon of TEA^+^. The photo-excitation energy is calculated using the HOMO-LUMO gap.

**Section S2**. Chemical analysis of H_2_BPDC-(NH_2_)_2_.


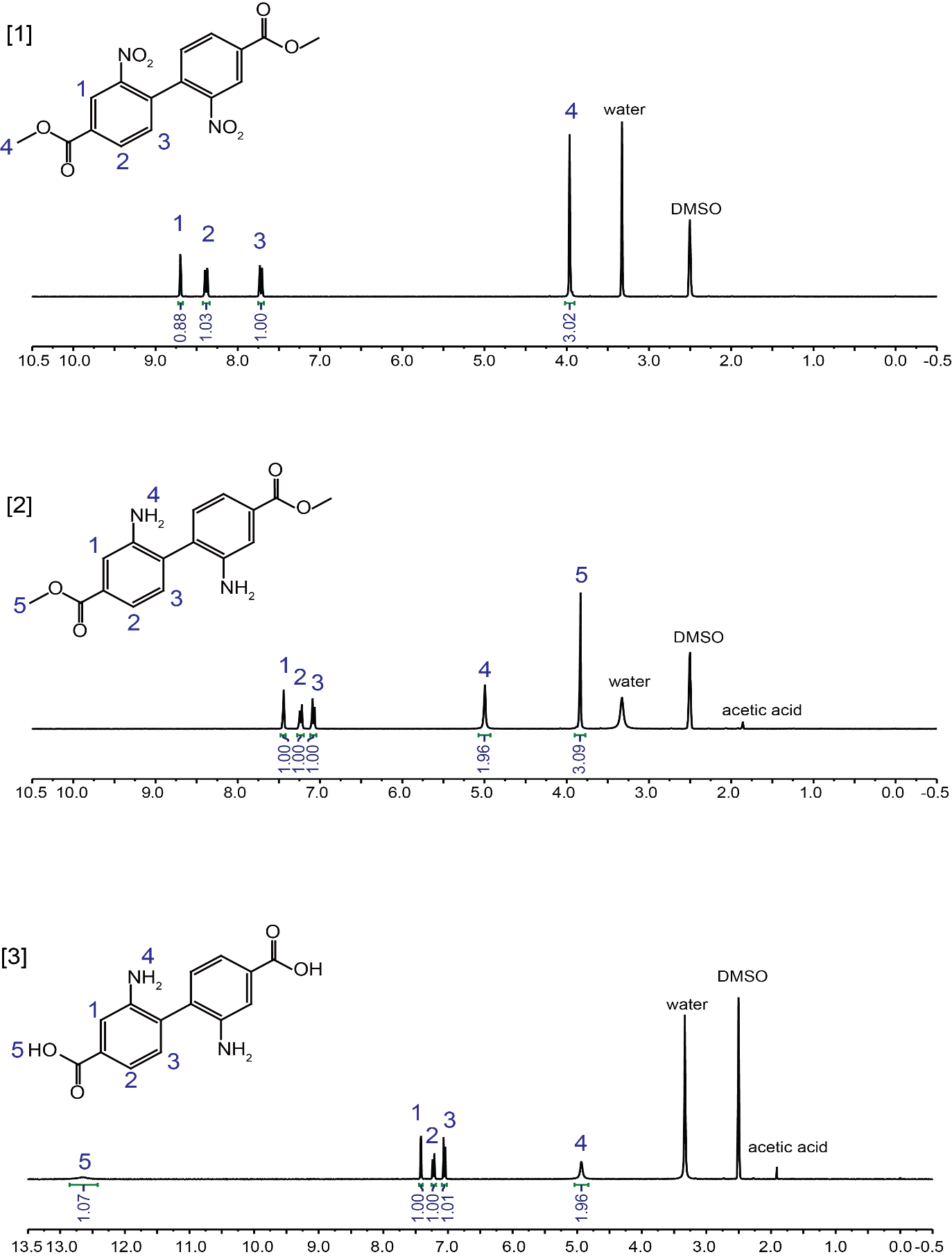


**Figure S1.** **^1^H NMR spectra for [1], [2], and [3].**

**Section S3.** Characterizations of Re-MOFs


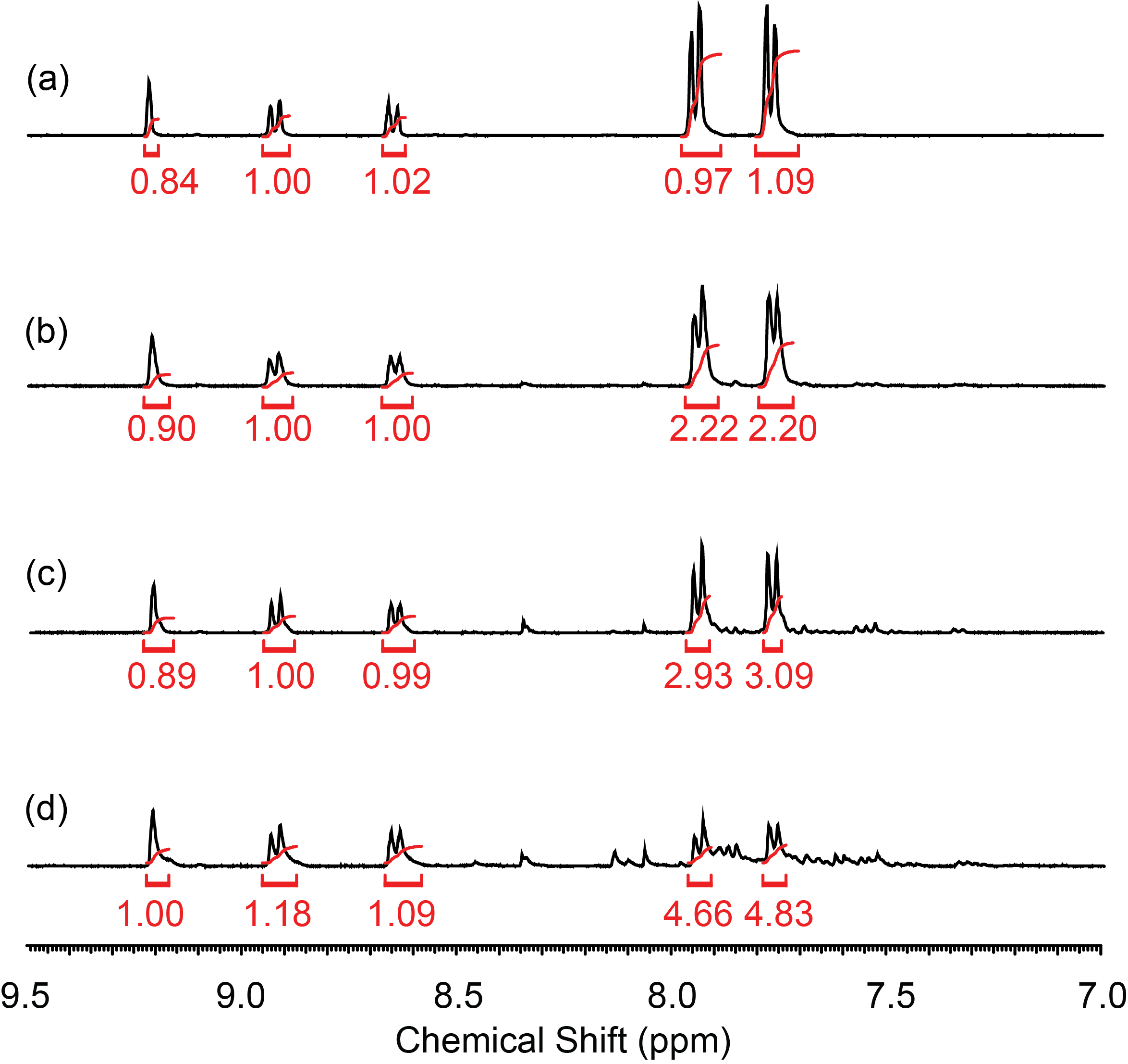


**Figure S2.** **Digest NMR spectra.** **(a)** Re-MOF, **(b)** Re-MOF-NH_2_(33%), **(c)** Re-MOF-NH_2_(52%) and **(d)** Re-MOF-NH_2_(64%). ^1^H-NMR spectrum was measured Re-MOF-NH_2_(X%) solutions dissolved in DCl/DMSO.


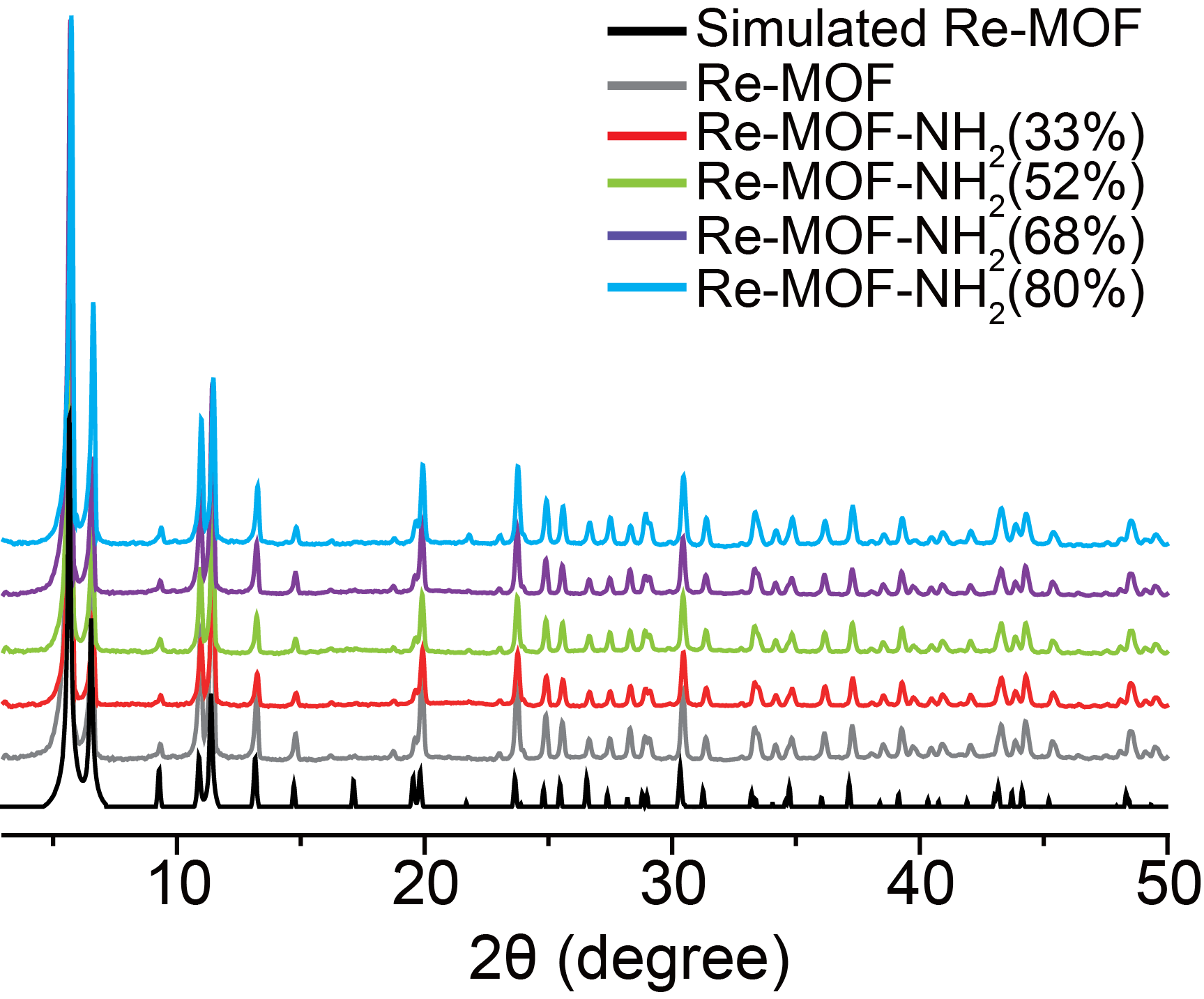


**Figure S3.** **PXRD patterns of Re-MOF-NH_2_(X%) with X=0, 33, 52, 68 and 80.**

**
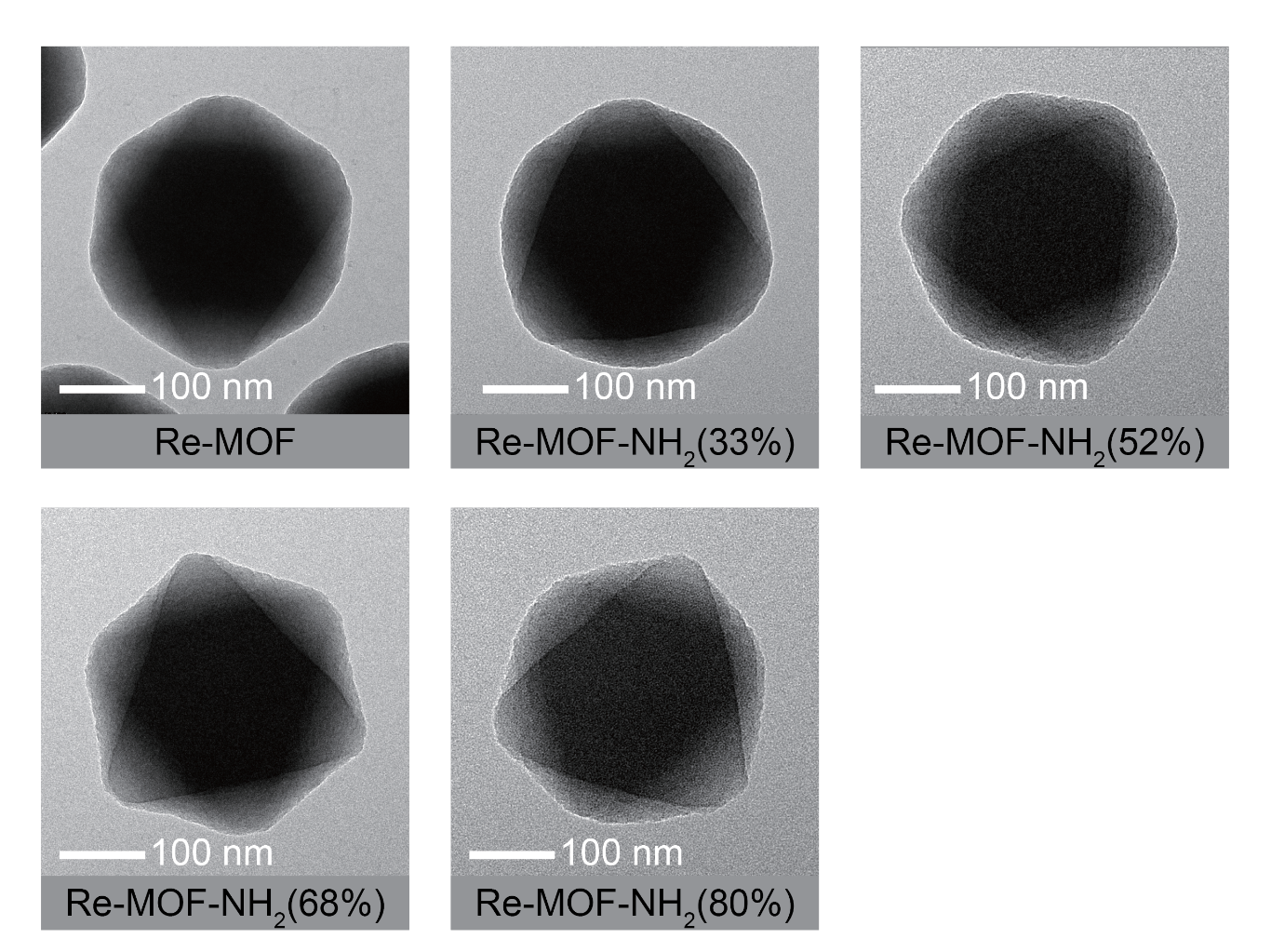
**

**Figure S4.** **TEM images of Re-MOF-NH_2_(X%) with** **X = 0, 33, 52, 68 and 80.**

**
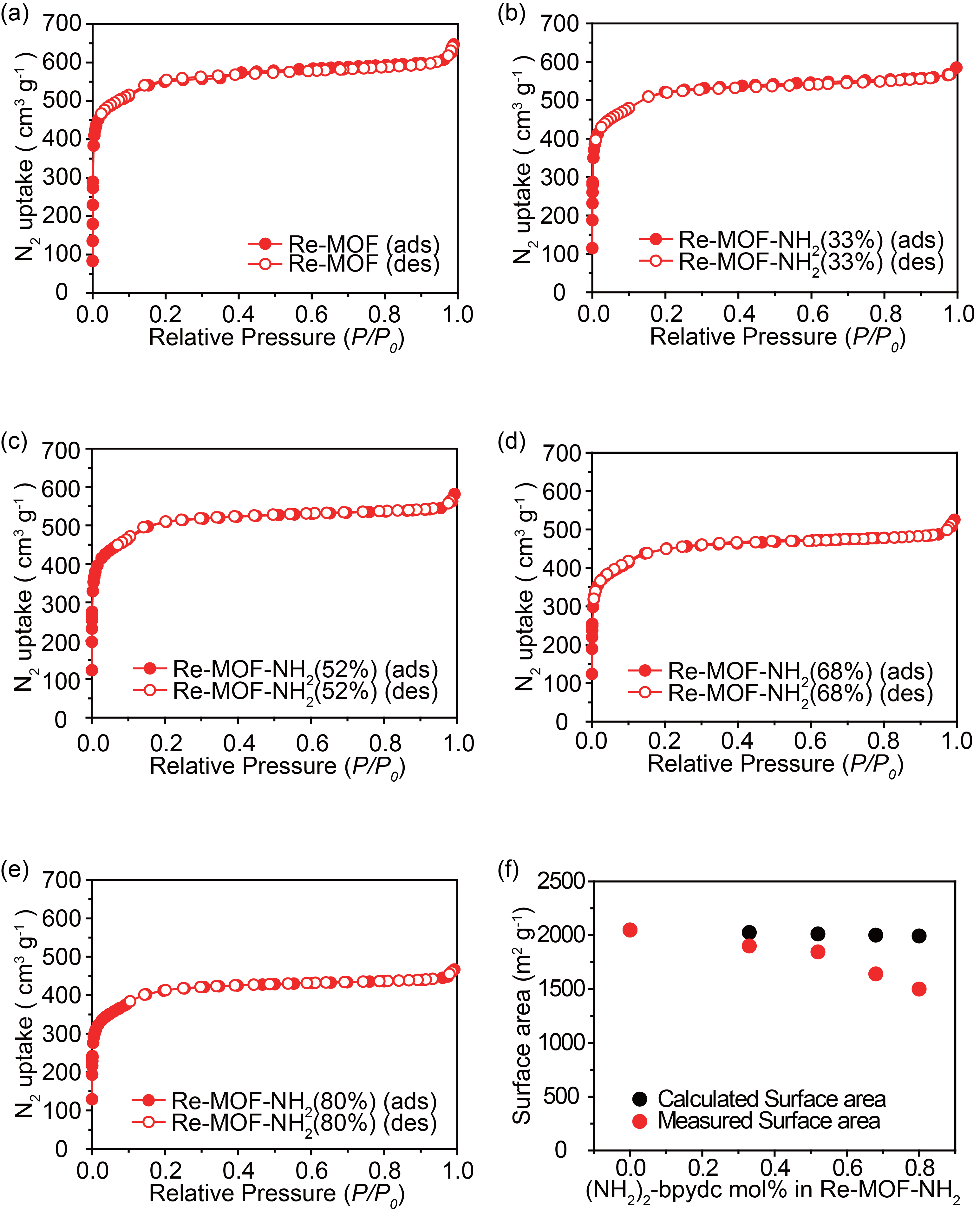
**

**Figure S5.** **N_2_ adsorption-desorption isotherms and corresponding Langmuir surface areas.** **(a-e)** N_2_ adsorption-desorption isotherms of Re-MOF-NH_2_(X%) at 77 K with adsorption and desorption points represented by closed circles and open circles, respectively (*P*/*P*_0_, relative pressure). **(f)** Langmuir surface area plot versus NH_2_ content for Re-MOF-NH_2_(X%).


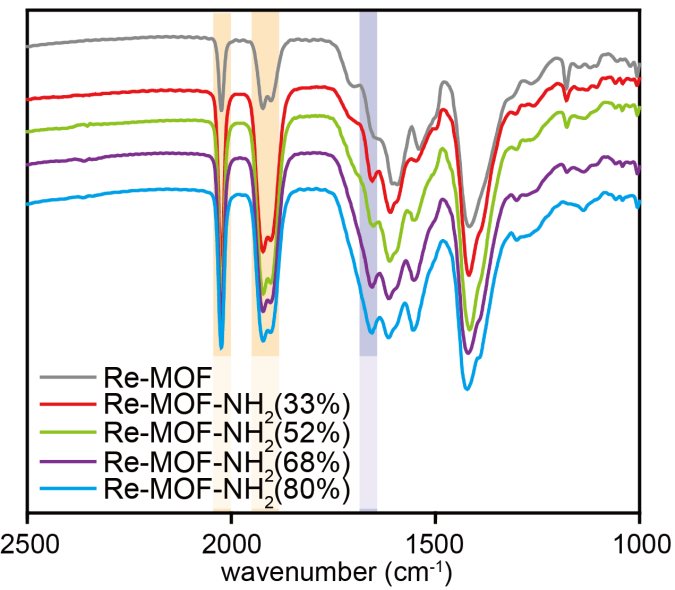


**Figure S6. IR spectra for Re-MOF and Re-MOF-NH_2_(X%) with X = 33, 52, 68, and 80.**


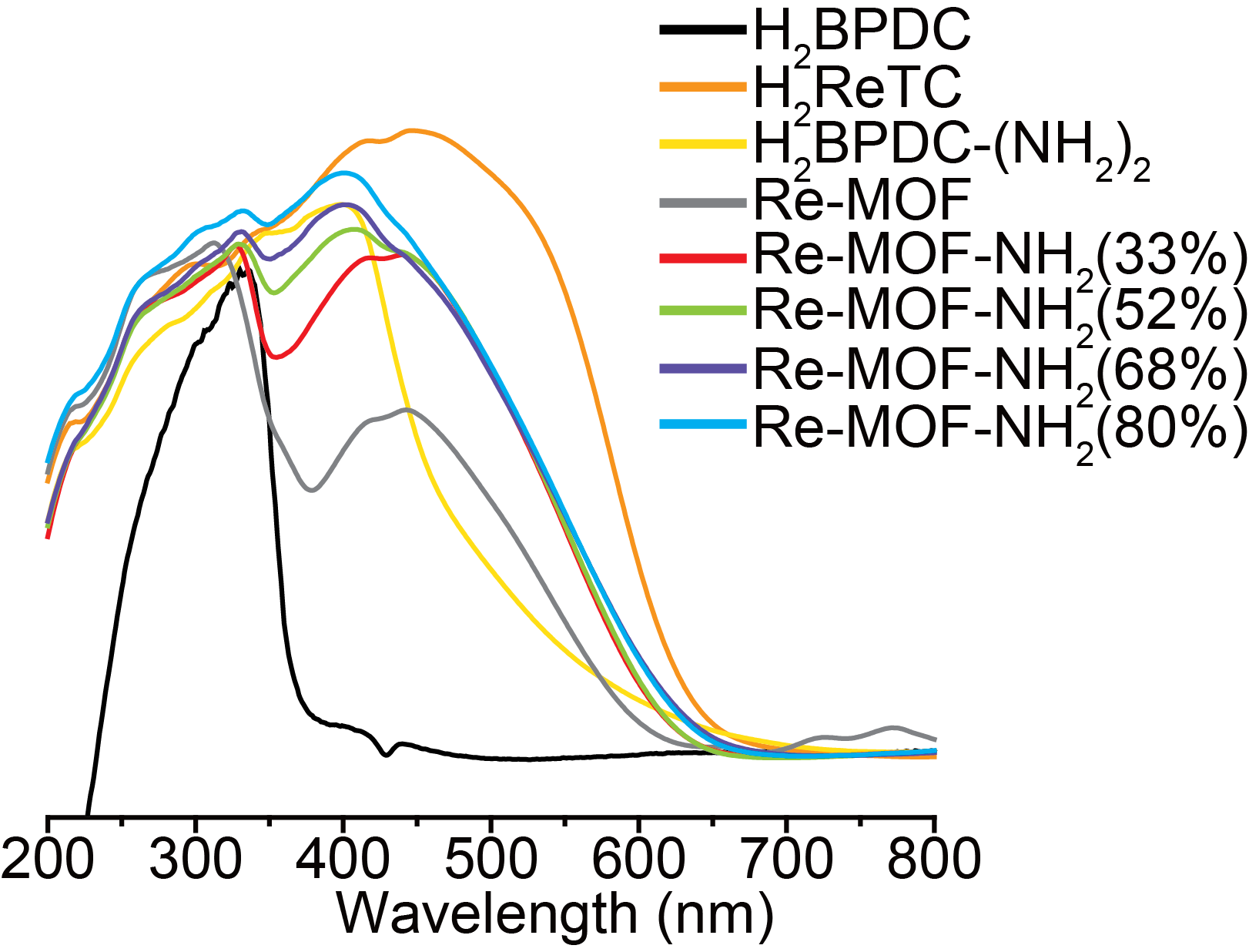


**Figure S7.** **UV-visible spectra of ligands (H_2_BPDC, H_2_ReTC, and H_2_BPDC-(NH_2_)_2_) and Re-MOF-NH_2_(X%) with X = 0, 33, 52, 68, and 80.**


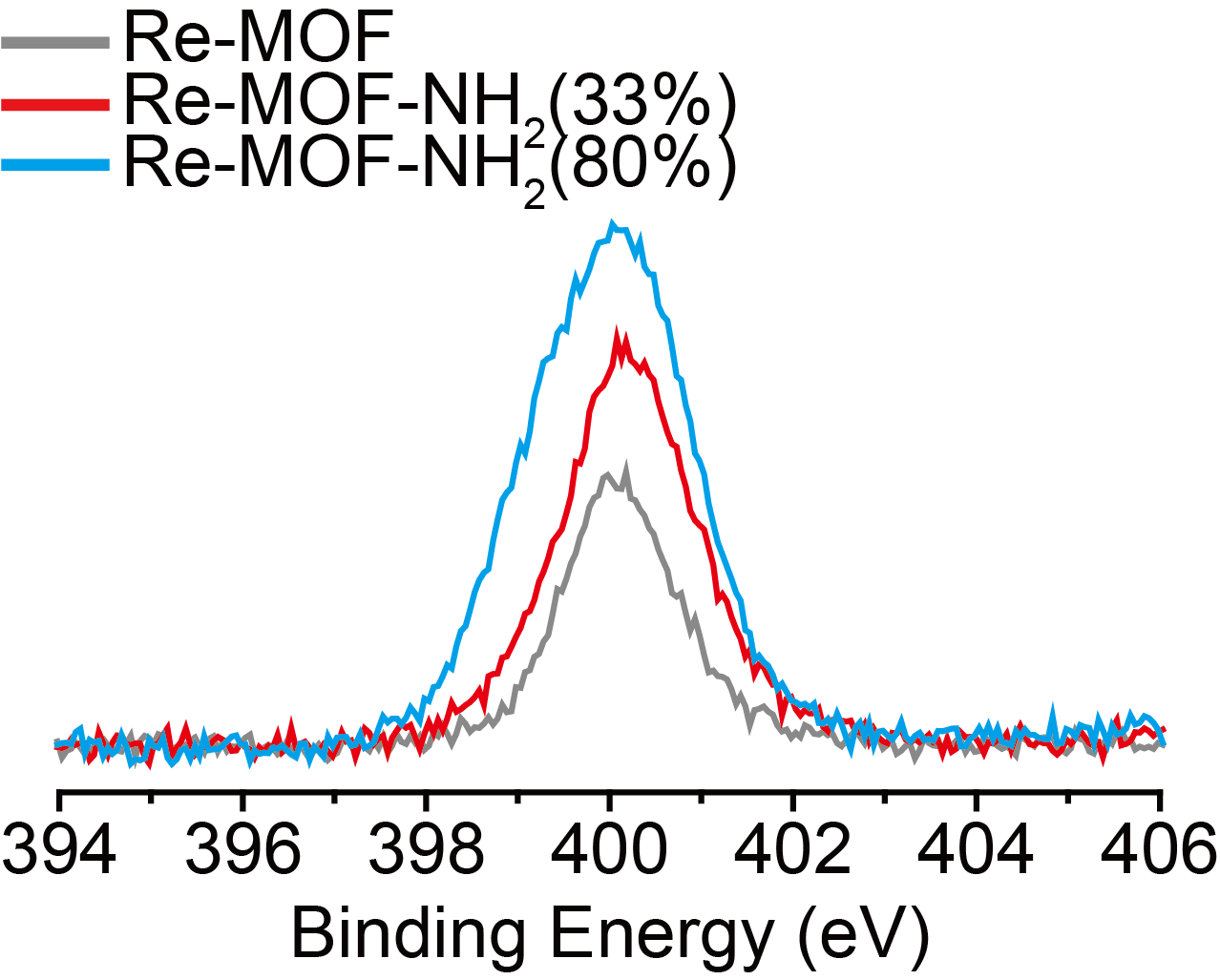


**Figure S8.** **XPS spectra for nitrogen contents in Re-MOF, Re-MOF-(NH_2_)_2_(33%) and Re-MOF-(NH_2_)_2_(80%).**


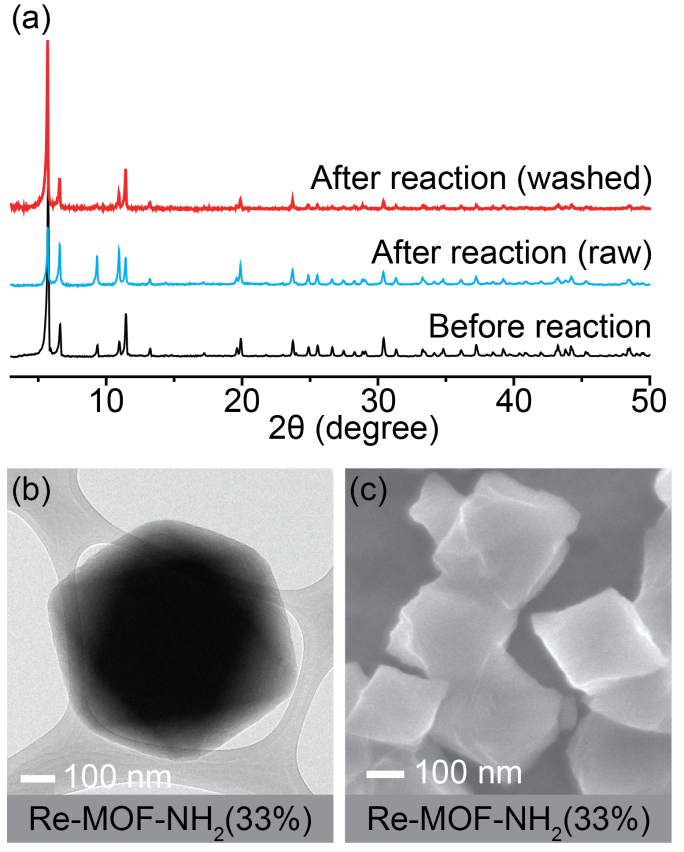


**Figure S9. TEM and SEM images of Re-MOF-NH_2_(33%).** **(a)** PXRD for Re-MOF-(NH_2_)_2_(33%) before and after reaction. **(b)** TEM image of Re-MOF-(NH_2_)_2_(33%) and **(c)** SEM image of Re-MOF-(NH_2_)_2_(33%) after the photocatalytic reaction


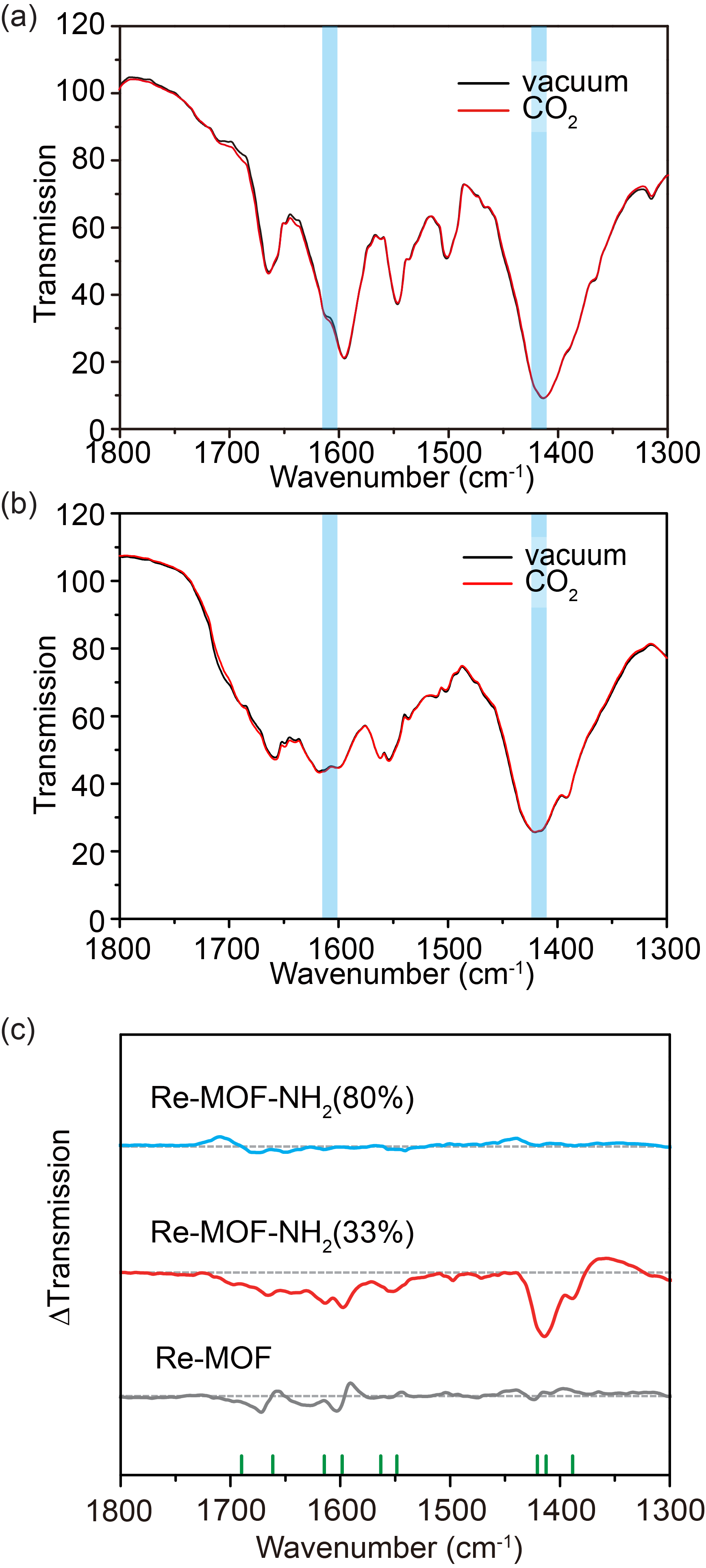


**Figure S10.** **Infrared (IR) of Re-MOF and Re-MOF-NH_2_(X%).** IR spectra for **(a)** Re-MOF and **(b)** Re-MOF-NH_2_(80%) in vacuum and CO_2_.


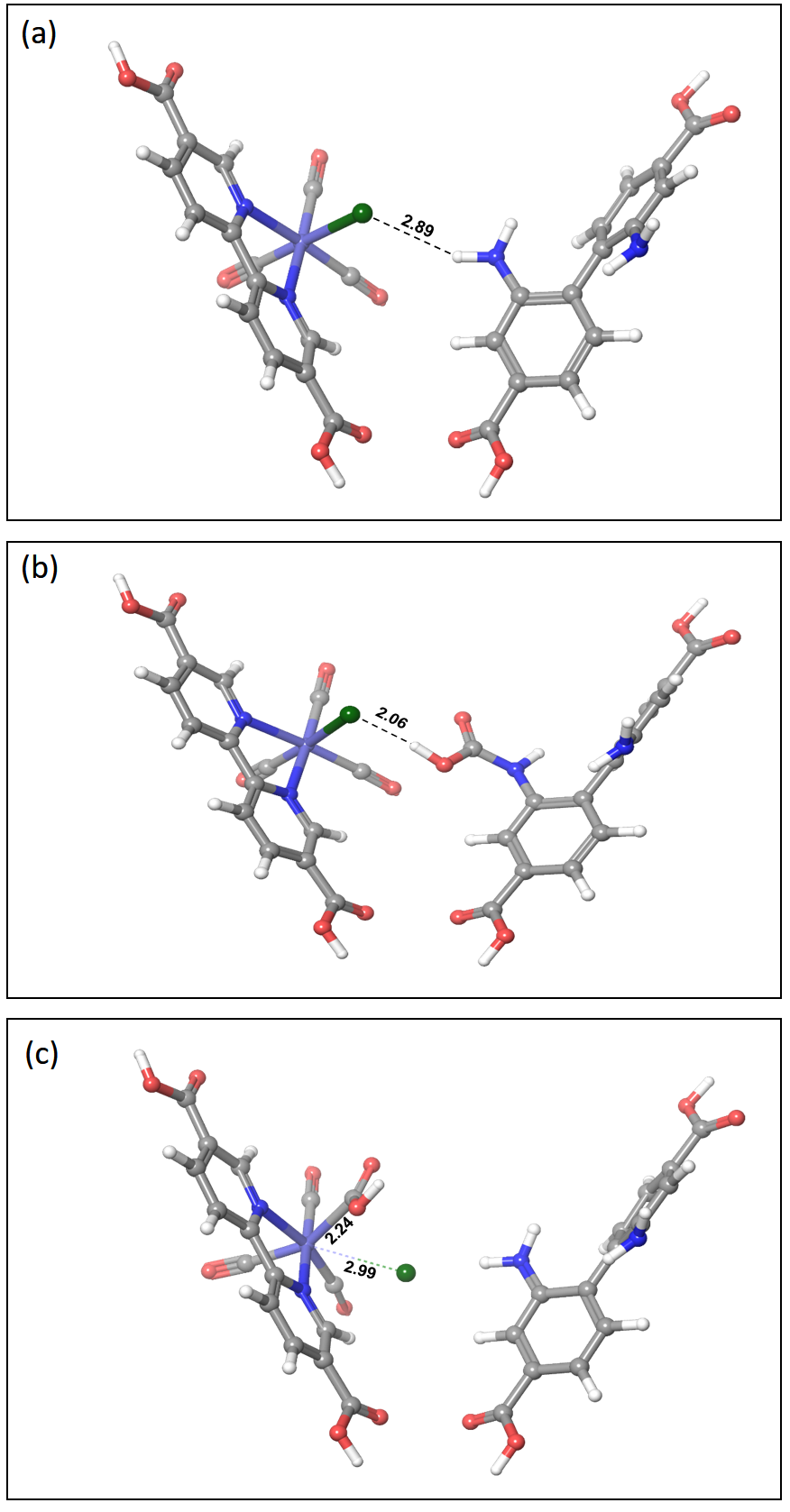


**Figure S11. Representative atomistic models determined from DFT calculations.** (**a**) The initial ReTC and –NH_2_ ligands, (**b**) the carbamate intermediate on –NH_2_ ligand, and (**c**) the 7-coordinate ReTC ligand with –COOH transferred from the carbamate originally formed in the –NH_2_ ligand, where C, N, O, and H atoms are represented by gray, green, red, and white spheres in color.


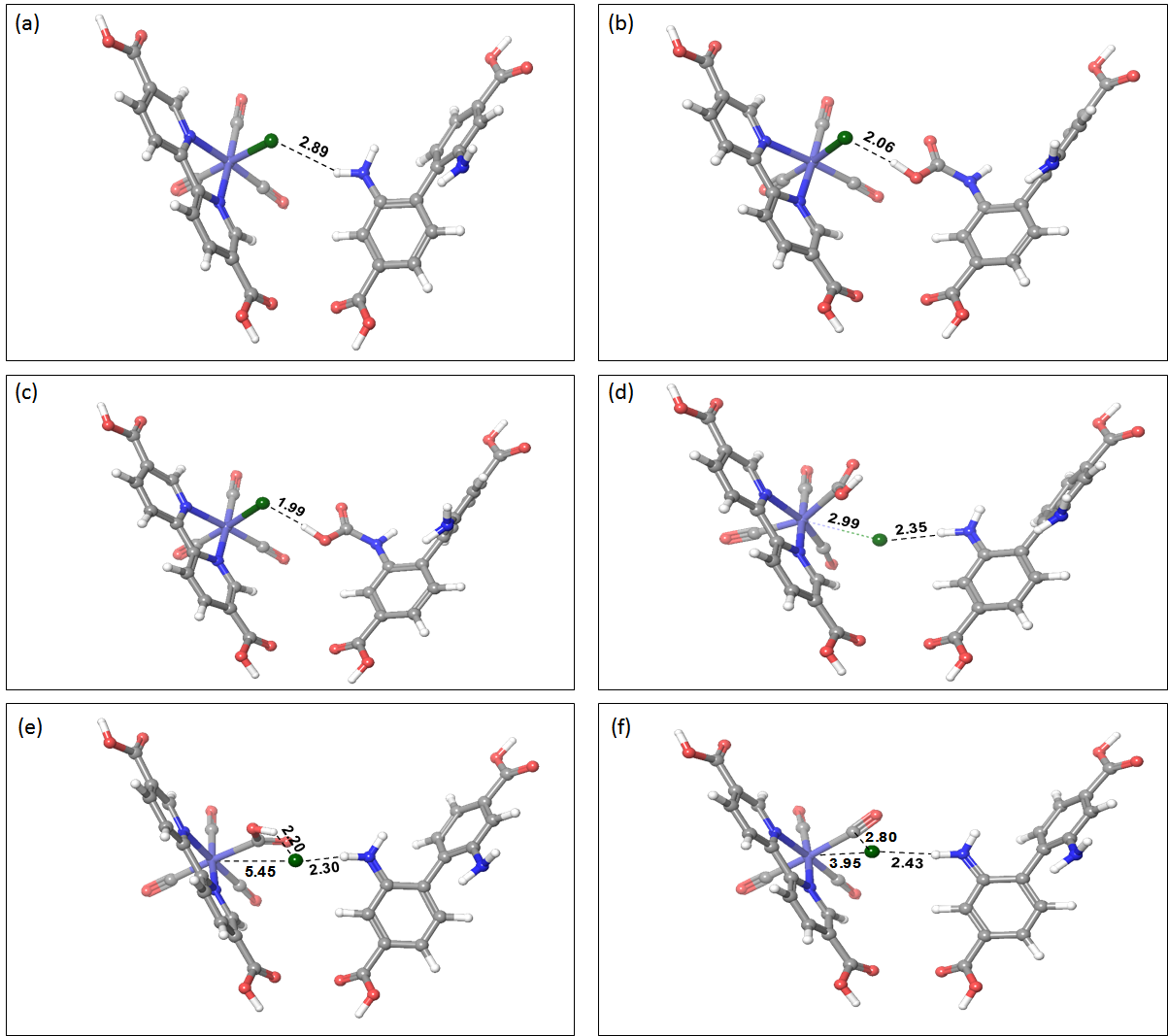


**Figure S12. Atomistic structures for catalyst active centers determined from DFT calculations.** (**a**) The initial ReTC and –NH_2_ ligands, (**b**) the carbamate formation on –NH_2_ ligand, (**c**) the structure after first electron uptake, (d) the 7-coordinate ReTC ligand with –COOH transferred from carbamate, (e) the structure after second electron uptake, (f) the final product CO adsorbed on ReTC, where C, N, O, and H atoms are represented by gray, green, red, and white spheres in color.

|  | ReTC | BPDC | BPDC-(NH_2_)_2_ |
| --- | --- | --- | --- |
| Re-MOF | 27 | 73 | - |
| Re-MOF-NH_2_(33%) | 27 | 40.5 | 32.5 |
| Re-MOF-NH_2_(52%) | 24 | 24 | 52 |
| Re-MOF-NH_2_(68%) | 21 | 10.5 | 68.5 |
| Re-MOF-NH_2_(80%) | 20 | 0 | 80 |

**Table S1.** **The contents of ReTC, BPDC and BPDC-(NH_2_)_2_ in Re-MOF-NH_2_(X%)** **with X corresponding to 0, 33, 52, 68, and 80.** These data were obtained through combined measurements of the inductively coupled plasma atomic emission spectroscopy (ICP-AES) for ReTC and the digest-^1^H NMR spectra for BPDC.

| Type | Photocatalyst | Main product  (μmol/g/h) | Experimental  details | Reference |
| --- | --- | --- | --- | --- |
| Organic | Re-MOF | CO(0.5) | Visible light | This work |
| Organic | Re-MOF-NH_2_(30%) | CO(1.5) | Visible light | This work |
| hybrid | g-C3N4/UiO-66 | CO(0.9) | Visible light | Reference S6 |

**Table S2.** **Performance comparison of the Re-MOF-NH_2_(X%) with that of a hybrid catalyst.**

**Section S4.** References

1. Ziessel R. Photocatalysis. Mechanistic Studies of Homogeneous Photochemical Water Gas Shift Reaction Catalyzed under Mild Conditions by Novel Cationic Iridium(III) Complexes. *J. Am. Chem. Soc*. **115**, 118–127 (1993).
2. Ko N. *et al.* A Significant Enhancement of Water Vapour Uptake at Low Pressure by Amine-Functionalization of UiO-67. *Dalton Trans*. **44**, 2047-2051 (2015).
3. Bochevarov A. D. et al., Jaguar: A High-Performance Quantum Chemistry Software Program with Strengths in Life and Materials Sciences. *Int. J. Quantum Chem*. **113**, 2110-2142 (2013).
4. Yanai,T., Tew D. P., Handy N. C. A New Hybrid Exchange–Correlation Functional Using the Coulomb-Attenuating Method (CAM-B3LYP). *Chem. Phys. Lett*. **393**, 51-57 (2004).
5. Hay P. J., Wadt W. R. Ab Initio Effective Core Potentials for Molecular Calculations: Potentials for K to Au Including the Outermost Core Orbitals. *J. Chem. Phys*. **82**, 299-310 (1985).
6. Shi L., Wang T., Zhang H., Chang K., Ye J. Electrostatic Self-Assembly of Nanosized Carbon Nitride Nanosheet onto a Zirconium Metal–Organic Framework for Enhanced Photocatalytic CO_2_ Reduction. *Adv. Func. Mat*. **25**, 5360-5367 (2015).
